# Partitioning the Human Transcriptome Using *HKera*, a Novel Classifier of Housekeeping and Tissue-Specific Genes

**DOI:** 10.1371/journal.pone.0083040

**Published:** 2013-12-20

**Authors:** Austin W. T. Chiang, Grace T. W. Shaw, Ming-Jing Hwang

**Affiliations:** 1 Bioinformatics Program, Taiwan International Graduate Program, Institute of Information Science, Academia Sinica, Taipei, Taiwan; 2 Institute of BioMedical Informatics, NationalYang-MingUniversity, Taipei, Taiwan; 3 Institute of Biomedical Sciences, Academia Sinica, Taipei, Taiwan; New Jersey Institute of Technology, United States of America

## Abstract

High-throughput transcriptomic experiments have made it possible to classify genes that are ubiquitously expressed as housekeeping (HK) genes and those expressed only in selective tissues as tissue-specific (TS) genes. Although partitioning a transcriptome into HK and TS genes is conceptually problematic owing to the lack of precise definitions and gene expression profile criteria for the two, information whether a gene is an HK or a TS gene can provide an initial clue to its cellular and/or functional role. Consequently, the development of new and novel HK (TS) classification methods has been a topic of considerable interest in post-genomics research. Here, we report such a development. Our method, called *HKera*, differs from the others by utilizing a novel property of HK genes that we have previously uncovered, namely that the ranking order of their expression levels, as opposed to the expression levels themselves, tends to be preserved from one tissue to another. Evaluated against multiple benchmark sets of human HK genes, including one recently derived from second generation sequencing data, *HKera* was shown to perform significantly better than five other classifiers that use different methodologies. An enrichment analysis of pathway and gene ontology annotations showed that *HKera*-predicted HK and TS genes have distinct functional roles and, together, cover most of the ontology categories. These results show that *HKera* is a good transcriptome partitioner that can be used to search for, and obtain useful expression and functional information for, novel HK (TS) genes.

## Introduction

Transcriptomics, which investigates patterns of gene expression across different tissues and different experimental conditions on a genome-wide scale, is a key component of post-genomics research. Genes that are ubiquitously expressed over a wide range of tissues and experimental conditions are usually called housekeeping (HK) genes, while those that are not are called tissue-specific (TS) or tissue-selective genes [1], [2]. To study a complex transcriptome, such as that of the human genome, it is often useful to determine which genes of the genome are HK genes and which are TS genes in order to understand their roles in cellular functions or disease processes [3]–[5]. Many bioinformatics tools have been developed for this purpose (e.g., [6]–[9]), although classification as HK and TS genes is not unambiguous, as it depends on the classification criteria and methodologies used [10].

At least six different methodologies have been used to partition a transcriptome into HK and TS genes, namely those that classify the genes based on the 1) magnitude of expression (*Exp*), 2) number of present calls of expression (*PCall*), 3) fraction present weighted expression intensity (*FPEI*), 4) tissue specificity index (*TSI*), 5) biophysical properties (*Phy*), or 6) Fourier analysis of expression data obtained at different time points in the cell cycle (*Fourier analysis*). *Exp* identifies genes as HK genes based on the criterion of high [2] or fairly constant [11], [12] expression, whereas *PCall* does not focus on the magnitude of expression, but, instead, uses a certain number of “present calls” as a threshold [13], [14], and *FPEI* [15] is a combination of the two. In contrast, *TSI* [16] uses a quantitative measure of variation in expression profiles in different tissues to evaluate the tendency of a gene to be HK (little tissue-wide variation) or TS (high variation). Different from all of the above, *Phy* [17], [18] ignores the expression data completely and uses the observations that, compared to TS genes, HK genes tend to be shorter [2], be flanked with more short repeats [17], have fewer protein domains [19], show lower promoter sequence conservation [20], and have simpler transcriptional regulation and slower rates of evolution [21], [22] to distinguish between HK and TS genes. Finally, *Fourier analysis* [23] transforms time-series gene expression data into Fourier spectra for a support vector machine (SVM, a machine learning method) to classify genes as HK or TS.

Despite their proven usefulness in numerous studies, all of these methods have shortcomings. For instance, *Exp*, *PCall*, *FPEI*, and *TSI* all tend to identify HK genes that are expressed at a high and/or fairly constant level and therefore may miss those expressed at a low level or at significantly different levels in different tissues [14], [24], [25]. As for *Phy*, despite the appeal of not using expression data, there have been conflicting results for the properties of HK genes (e.g. whether the gene structures of HK genes are compact [2], [26] or not [27]), which is not surprising, since there is a significant overlap in static gene properties between HK and non-HK genes [28]. Finally, the main limitation of the more sophisticated *Fourier analysis* is its use of time-series expression data and the resultant higher cost.

We have previously shown that the ranking order of expression levels for HK genes tends to be preserved from one tissue to another and that dispersion, stableness, and co-expression are the three factors making the greatest contribution to this novel property of HK genes that can be decomposed into a composite of 16 tensor components [28]. Here, we describe the development of an SVM classifier for designating a given human gene as an HK or TS gene based on the tensor structure of tissue-wide gene expression profiles. We have named this classifier *HKera*, ‘era’ being an abbreviation for ‘expression ranking assessment’. *HKera* is similar to *Fourier analysis* in that they both utilize a mathematical transformation of an underlying structure of gene expression data, but *HKera* does not require time-series data.

To evaluate the performance of an HK gene classifier, a so-called ‘gold-standard’ set of HK genes is required, and several such sets have been derived and used as the benchmark to evaluate HK (TS) gene classifiers [2], [6], [10], [14]. In this work, *HKera* and five other HT (TS) prediction methods were evaluated using three widely-used HK gene sets and one very large HK set derived recently from RANseq experiments as benchmark. The results showed that *HKera* performed significantly better than the five other methods evaluated (a comparison with *Fourier analysis* was not made because we did not use time-series data). Furthermore, an analysis using the functional annotations of the Kyoto Encyclopedia of Genes and Genomics (KEGG) [29], Protein Information Resource (PIR) [30], and Gene Ontology (GO) [31] revealed that functional categories enriched in HK genes are distinct from those enriched in TS genes, supporting the notion that, by and large, the two have distinct functional roles in the cell.

## Materials and Methods

### Datasets

The GSE2361 Affymetrix microarray data for human genes compiled by Ge et al. [32] was downloaded from GEO depositories [33] and processed using previously described procedures [28]. This GSE2361 dataset contains gene expression profiles for 13,075 genes in 36 normal human tissues. As previously [28], this dataset was divided into three gene sets, namely HK, TS, and MR (“middle-ranged”), the HK set consisting of a set of 388 genes present in the 408 HK gene set manually curated by Zhu et al. [10], the TS set consisting of 734 genes that satisfy four stringent criteria for being TS genes [28], [32], and the MR set consisting of the remaining 11,953 genes. Three hundred HK genes and 300 TS genes were then randomly selected from the HK or TS set to train and test the SVM models of *HKera* described below.

We treated the set of 388 HK genes curated by Zhu et al. [10] and two other HK sets derived from microarray data [2], [14] as ‘gold-standard’ HK genes, since they have been used as such in the past (e.g. [15], [18], [28]). The HK set identified by Ramskold et al. [6] was also treated as gold-standard, since it was derived from RNAseq experiments, which can detect expression signals more comprehensively and at a higher resolution than conventional microarray experiments [34]. These four HK gene sets were denoted, respectively, as HK_388_, HK_383_, HK_557_, and HK_6121_, the subscript indicating the size (number of genes with expression data in GSE2361) of the set, while the set of 734 TS genes used to train/test *HKera* was denoted as TS_734_.

### Development of *HKera*

**Figure 1:**
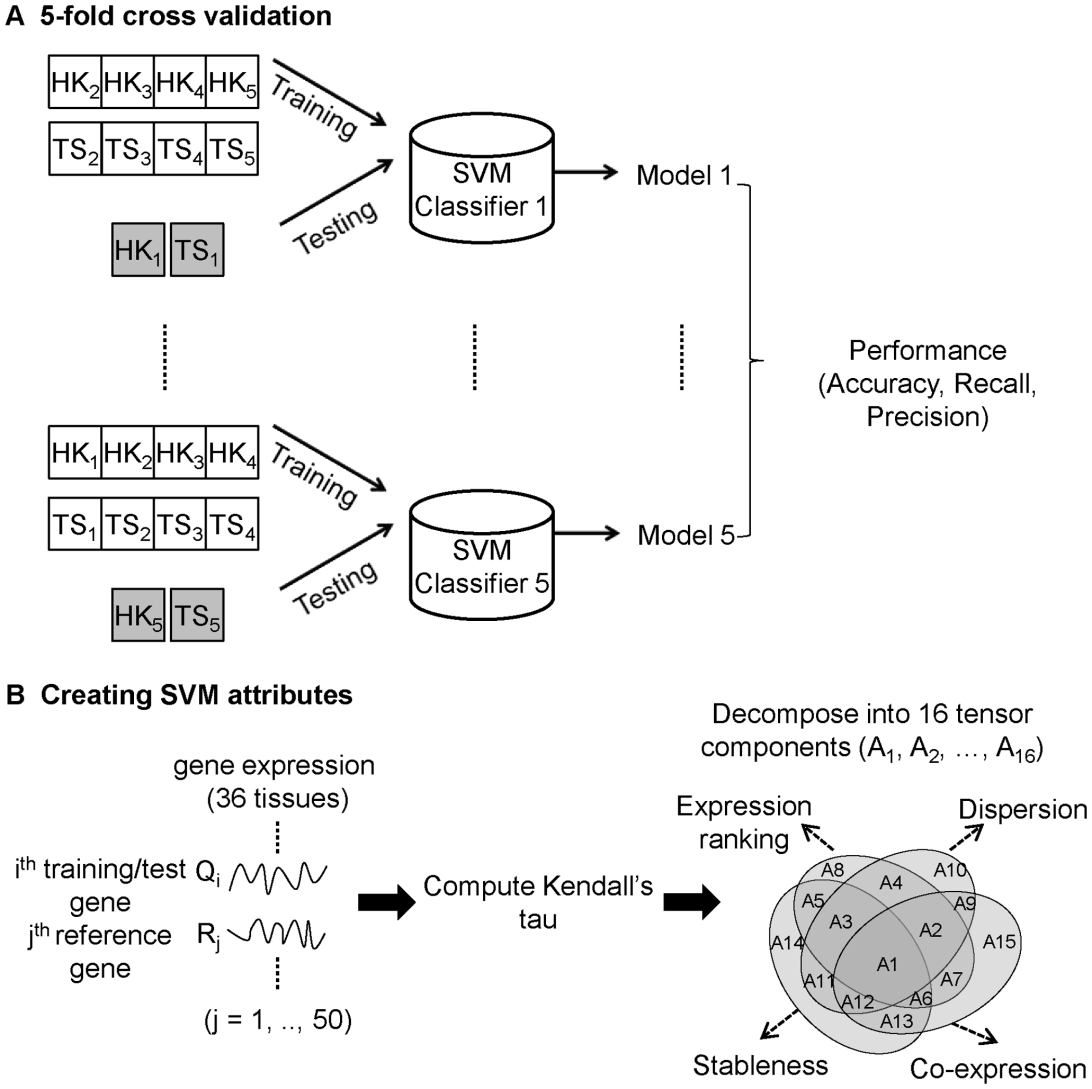
Developing the *HKera* classifier. (A) The *HKera* classifier is one of five SVM models resulting from 5-fold cross validation of SVM learning on 300 ‘gold-standard’ HK genes and 300 ‘gold-standard’ TS genes (see Methods). (B) To create the attributes for the SVM learning, each query gene used for training and test (Qi) was paired with each of 50 reference genes (Rj) (see Methods) to compute the Kendall’s 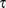 of their tissue-wide gene expression profile, which was then decomposed into 16 tensor components (A1, A2, …, A16) following our previously described procedure [28]. The mean of each tensor component averaged over a set of query genes (e.g. HK1, TS1, etc. in (A)) provided one of the16 attributes used to train/test the SVM models.

*HKera* is an SVM model from a five-fold cross validation, in which the 300 HK (and 300 TS) genes randomly selected from the HK_388_ and TS_734_ sets were arbitrarily partitioned into five subgroups of equal size, each of which was alternately used for testing, while the remaining four subgroups were used for training (Figure 1A). This resulted in five models and, although fairly similar results were obtained for all five (see Results), for simplicity, the one with the best numerical performance was chosen as the *HKera* classifier.

The attributes used to train the SVM models were the 16 components (Figure 1B) derived by tensor decomposition of Kendall’s τ, a measure of tissue-wide concordance in the ranking order of expression levels between any two genes [28]. The meanings of these 16 attributes are schematically illustrated and explained in supplemental Figure S1 in File S1, along with an example of actual data for a specific gene pair in Figure S2 in File S1. Since this method requires data for gene pairs, it was necessary to have a set of reference genes with which to pair any query gene (training or testing). In principle, any gene can serve as a reference gene. Indeed, similar performances were obtained when three very different reference gene sets were used (see supplemental Table S1 in File S1). These three reference gene sets contained, respectively, 50 HK genes, 50 TS genes, or 25 HK and 25 TS genes randomly selected from the HK_388_ and TS_734_ sets described above after excluding those already selected to be included in the training and test sets. To demonstrate it was not necessary to use only HK genes as reference genes to derive *HKera*, in this study, we report only the results of the *HKera* built using the reference set of 50 TS genes. We employed the bioinformatics toolbox of the Matlab software (version 7.6.0.324, release R2008a) [35] available at http://www.mathworks.com, particularly its “*svmdecision*” command, to build the *HKera* classifier. Using *HKera*, every gene in the training set received a score from −1 to +1, indicating the extent of its tendency to be an HK gene (a more positive score) or a TS gene (a more negative score), but some test genes may receive a score slightly beyond the −1 or the +1 limit. In this work, those with a positive score were regarded as HK genes and those with a negative score as TS genes. Using a threshold of zero, the GSE2361 data set (13,075 genes) was partitioned into 8,072 HK genes (61.7%) and 5,003 TS genes (38.3%).

### Performance Evaluation

To evaluate *HKera* and other HK prediction methods, we calculated the following performance measures:
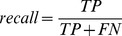
(1)

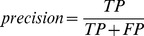
(2)

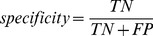
(3)

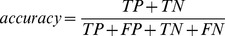
(4)where *TP* denotes true positive, *FP* false positive, *TN* true negative, and *FN* false negative. When evaluating the five-fold cross validation, *TP* was the number of correctly predicted HK genes for the 300 genes chosen from the HK_388_ set to be included in the training and testing sets and *TN* was the number of correctly predicted TS genes for the 300 genes chosen from the TS_734_ set; when computing the receiver operating characteristic (ROC) curves [36] for *HKera* and the other methods to compare their performance, *TP* was the number of correctly predicted HK genes in each of the four benchmark HK sets, i.e. HK_388_ [10], HK_383_ [14], HK_557_ [10], and HK_6121_ [6], and *TN* was the number of correctly predicted TS genes in the TS_734_ set. For both computations, *FP* was the number of TS_734_ genes predicted to be HK and *FN* the number of benchmark HK genes predicted to be TS. A few genes present in both the TS_734_ set and the benchmark HK set (3 using HK_383_, 5 using HK_557_, and 103 using HK_6121_) were excluded from the computation of the performance measures. The criteria used to define HK genes in the GSE2361 dataset using the various methods compared in this study are listed in Table 1.

**Table 1:** HK criterion and the resulting number of HK genes in the GSE2361 set using different methods.

Method	HK criterion	Number ofHK genes	Reference
*Exp*	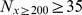 a	1,114	[2]
*PCall*	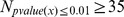 b	1,685	[13], [14]
*FPEI*	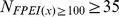 c	2,064	[15]
*TSI*	*TSI 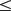 *0.1^d^	1,039	[16]
*Phy*	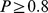 e	1,219	[18]
*RNAseq*	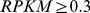 f	6,121	[6]
*HKera*	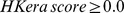	7,761	This work

a Any gene is an HK gene if it has an expression intensity (*x*) > = 200, as recommended by [2], in at least 35 tissues. *N* is the number of tissues.

b The *p* value for gene expression intensity needs to be less than 0.01 to make a detection call of ‘Present’ [13], and a gene needs to have a ‘Present’ call in at least 35 tissues to be considered an HK gene. *N* is the number of tissues.

c Following [15], genes with an *FPEI* score above 100 in at least 35 tissues were defined as HK genes. *N* is the number of tissues.

d *TSI* [16] is bounded between 0 and 1. A lower *TSI* indicates a lower tendency for the gene to be TS (or a higher tendency for it to be HK). The 0.1 threshold was chosen following [16].

e For each gene, the Näive Bayes classifier [18] calculates a probability (*P*) of it being an HK gene; in this study, we choose those with a *P* value greater than 0.8 to be classified as HK genes.

f According to [6], those genes with an *RPKM* (reads per kilobase of exon model per million mapped reads) score greater than 0.3 were classified as HK genes.

### Functional Annotation and Enrichment Analysis

About two-thirds of the MR genes were scored as positive by *HKera*, yielding thousands of predicted HK genes. To investigate what functional roles of these genes might differ from those of the genes scored negatively and thus classified as TS genes, genes of the MR set were sorted by their *HKera* score and divided into four sets of putative HK genes and two sets of putative TS genes, each containing about 2,000 genes. The choice of 2,000 as a cut-off was arbitrary, but was based on the consideration that a choice of 1,000 would result in each group having too few members for enrichment analysis (data not shown). We employed the DAVID Bioinformatics Resource [37], [38] to compute the p value for genes in each set and for genes in the HK_388_ set and the TS_734_ set to be associated with a specific pathway category of KEGG [29] and the p value for the likelihood of their being ubiquitous according to PIR [30]. We then extended the enrichment analysis to GO categories [31], in which comparisons were made with gene sets derived from *FPEI* [15] and RNAseq experiments [6].

## Results

### Performance of SVM Models

Table 2 summarizes the performance for each of the five SVM models resulting from five-fold cross validation on training/test data of randomly selected HK and TS genes (see Methods). The three measures (accuracy, recall, and precision) were nearly all >99% perfect for training and remained very good (the worst being 87% recall for one model) for the test. We chose model 3 to be *HKera* because it gave the best test result for all three performance measures. Note that although the 16 attributes used by *HKera* were derived from ranking order data [28], they themselves are not ordinal data and therefore machine learning methods for ordinal classifications [39]–[41] may not be easily applied, not to mention that, to our knowledge, these methods tend to classify data into ordinal classes (i.e. the output end of the learning) but do not classify data with ordinal features (i.e. the input end). When five other machine leaning methods (decision tree, neural network, rule learner, naïve Bayes, and instance-based learning algorithm) representing different categories of classification methodologies [42] were used instead of SVM, the results showed that *HKera* (i.e. SVM) was among the best performers (Figure S3 in File S1).

**Table 2:** Performance of *HKera*’s SVM models derived from 5-fold cross validation on training/test data.

	Training (%)			Test (%)		
Model	Accuracy	Recall	Precision	Accuracy	Recall	Precision
1	99.2	98.8	99.6	92.0	87.7	96.1
2	99.2	99.0	99.4	92.3	89.7	94.9
**3** *	**99.3**	**98.7**	**99.9**	**95.2**	**93.3**	**96.9**
4	99.5	99.3	99.8	91.7	87.3	95.8
5	99.0	98.2	99.7	93.5	90.0	96.9

* Model 3 was chosen to represent *HKera* in this study.

### Comparisons with Other Methods using Different Benchmark HK Sets

**Figure 2:**
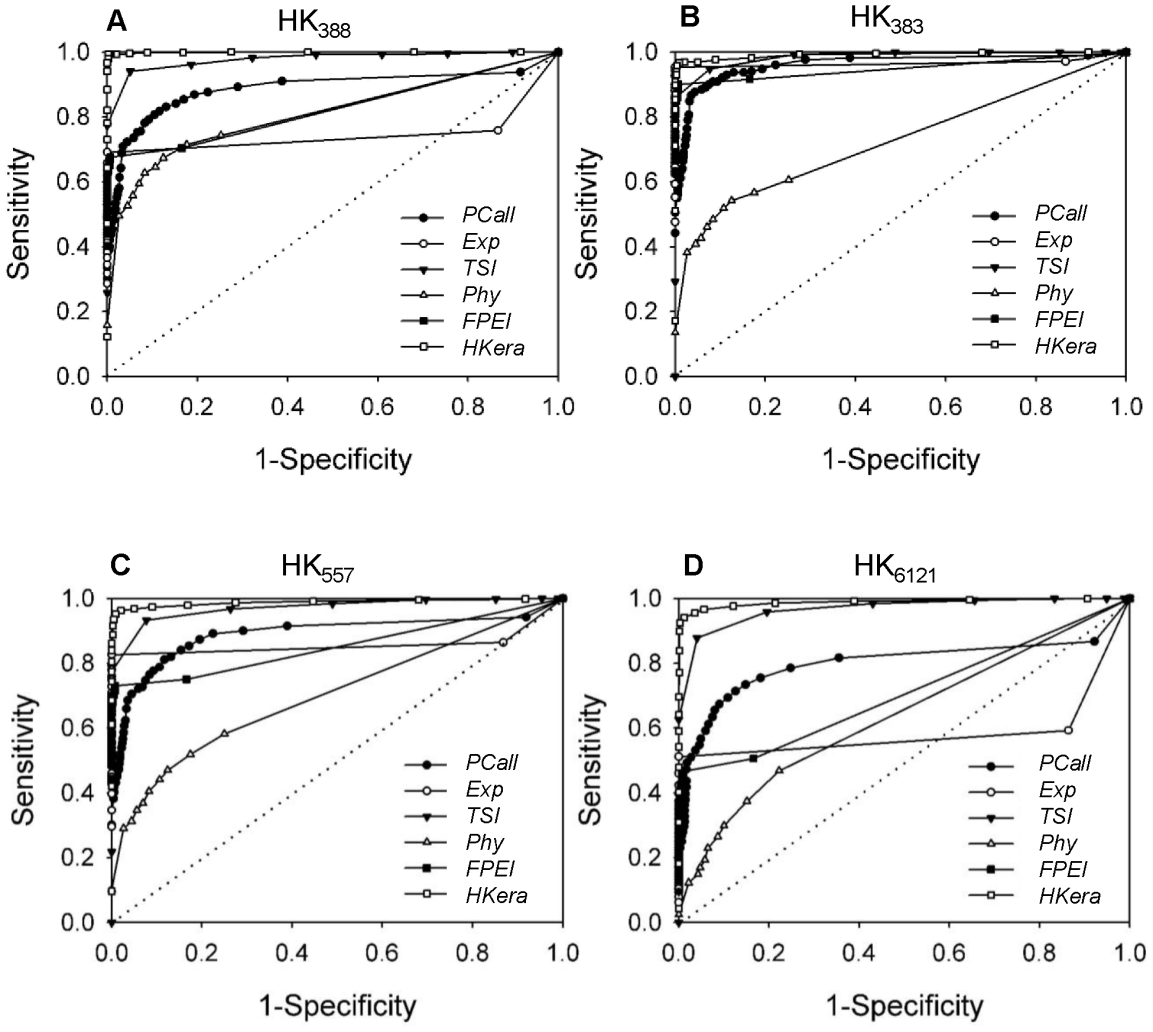
Performance of six HK prediction methods on four benchmark HK sets. The plots are ROC curves of sensitivity vs. 1-specificity, where sensitivity (i.e. recall) and specificity are defined, respectively, by Equation (1) and Equation (3) in the Methods. The six HK prediction methods compared are *PCall* [13], [14], *Exp* [2], *TSI* [16], *Phy* [18], *FPEI* [15], and *HKera* (this work), and the four benchmark HK sets are (A) HK_388_ [10], (B) HK_383_ [14], (C) HK_557_ [2], and (D) HK_6121_ (*RANseq*) [6].

The abilities of *HKera* and five other methods (*PCall*, *Exp*, *FPEI*, *Phy*, and *TSI*) to identify HK genes from the GSE2361 dataset were compared using the ROC evaluation of four benchmark HK sets (HK_388_ [10], HK_383_ [14], HK_557_ [10], and HK_6121_ [6]). ROC is a measure of sensitivity (i.e. recall ability) as a function of specificity, in which a larger area under the ROC curve (conventionally known as the AUC) indicates a better performance. As shown in Figure 2, when the sensitivity, or the percentage of recall, was not required to be high, most of the HK genes recalled were correct (i.e. specificity high, or 1-specificity close to zero) for all methods, but when greater sensitivity was required, differences in performance between the methods became apparent. With all sets, *HKera* exhibited the best performance, producing not only an almost perfect ROC curve for HK_388_ on which the classifier was trained (see Methods), but also an excellent ROC curve for the other three benchmark sets. Given its simplicity, *TSI*, which, like *HKera*, employs a mathematical transformation, albeit a much simpler one [16], performed surprisingly well. In contrast, *PCall*, *Exp*, and *FPEI* all exhibited an unbalanced performance, being accurate at low sensitivity, but bad at high sensitivity, except when using the HK_383_ set. Compared to *PCall*, *Exp*, and *FPEI*, *Phy* had a prediction accuracy that was relatively insensitive to the HK set used for evaluation, but a much smaller AUC, presumably owing to its use of static gene properties and not expression data, as mentioned above.

### *HKera* Scores and Coverage of Benchmark HK Sets

**Figure 3:**
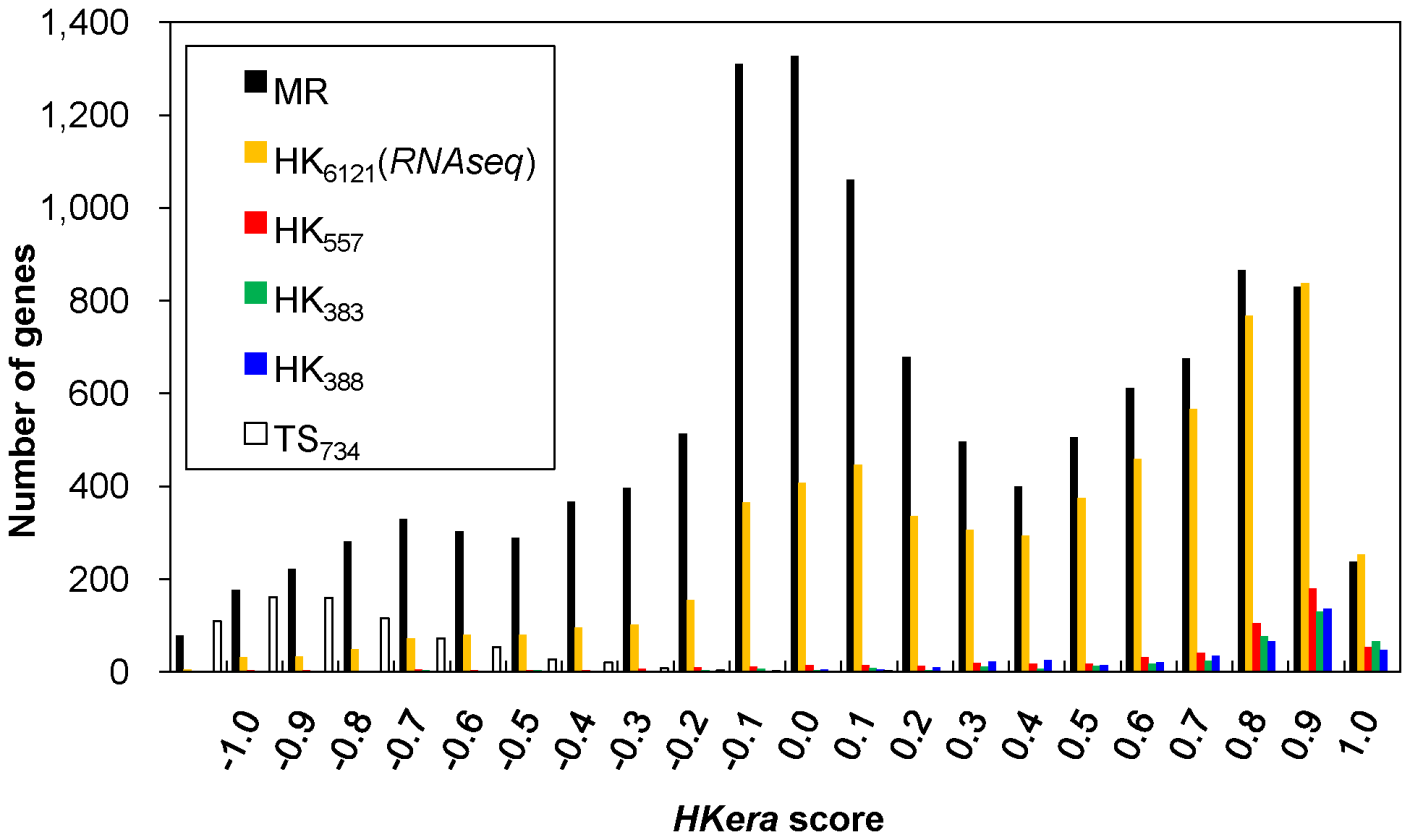
Distribution of *HKera* scores for various gene sets. Using the *HKera* classifier, every gene in each gene set was scored by a numerical value that indicated its tendency to be HK (more positive) or TS (more negative). Note that the entire GSE2361 set was scored, as it was divided into the three sets HK_388_, TS_734_, and MR (see Methods). In this work, 0.0 was the threshold used to divide the MR set into HK genes and TS genes.

**Figure 4:**
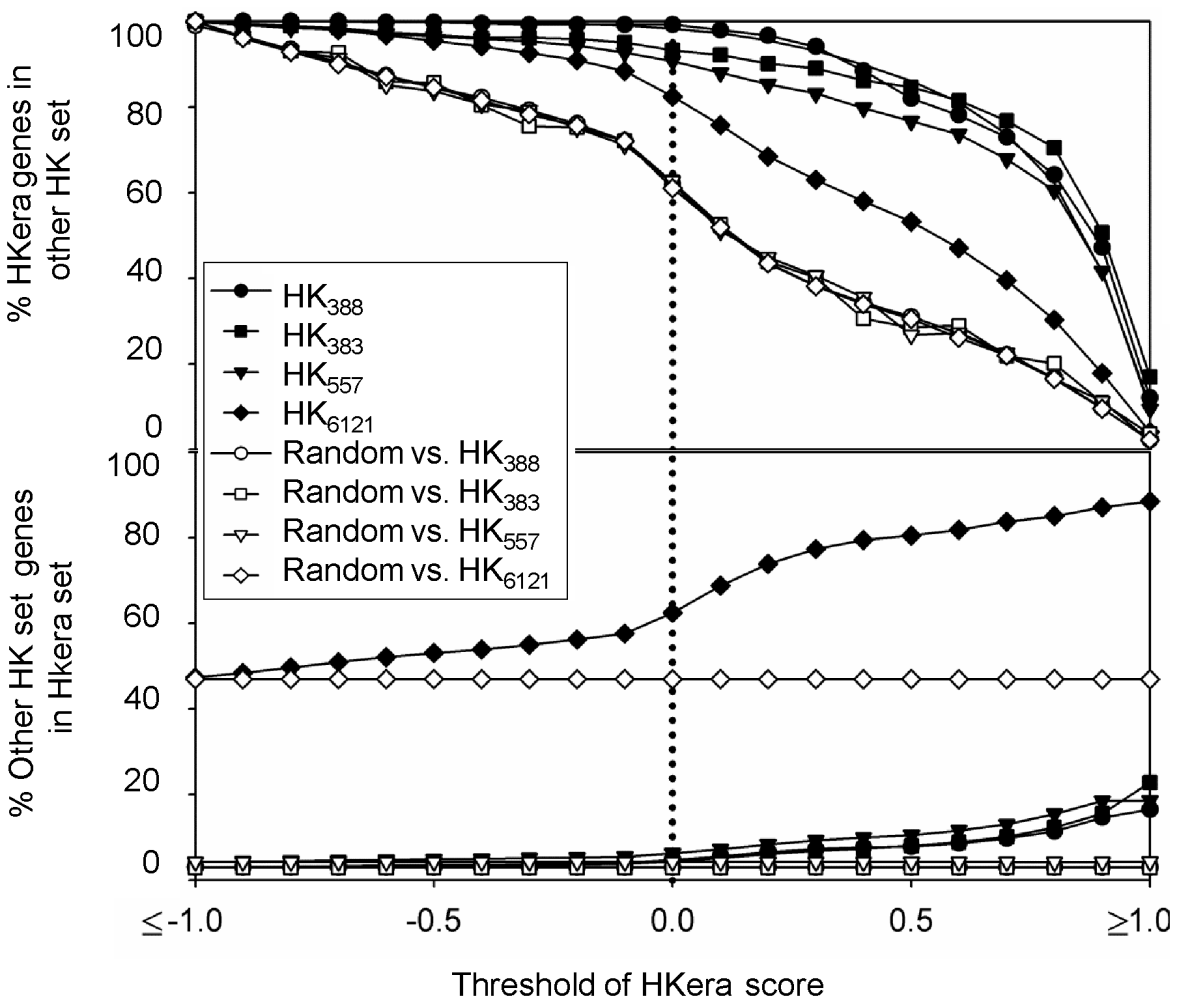
Cross-coverage between *HKera* and benchmark HK sets at various thresholds of the *HKera* score. At each score threshold, genes with a score greater than the threshold were classified as HK genes, while the same number of genes was randomly selected from the whole pool (GSE2361) to form a random set. The percentages of *HKera*-predicted HK genes (solid symbols) and randomly selected genes (empty symbols) that were present in the benchmark set considered were computed at various thresholds of the *HKera* score (top panel); the percentage of the reverse coverage was also computed (bottom panel).

Figure 3 shows the distribution of *HKera* scores using various benchmark gene sets and the MR set, which, together with HK_388_ and TS_734_, contain the entire 13,075 genes of the GSE2361 set. Overlaps of the *HKera* scores were seen among the various gene sets, including between the HK_388_ set, the TS_734_ set, and the MR set (11,953 genes), reinforcing the notion that HK and TS genes are largely distinguished based on qualitative descriptions and different quantitative measures will yield different HK/TS genes. Nevertheless, numerous MR genes appeared to have expression characteristics similar to those of expert-curated HK genes, as suggested by their similar high *HKera* scores, and can therefore be considered as HK genes in the subsequent functional analysis. Although a more positive *HKera* score indicates a higher tendency of having canonical HK expression characteristics, 0.0 was chosen as the threshold to partition the human transcriptome (Figure 3), because it produced a balanced cross-coverage between *HKera*-predicted HK genes and those determined from RNAseq data (Figure 4). An Excel file containing a complete listing of the 13,075 GSE2361 human genes ordered by *HKera* score is provided as a supplement (Table S2 in File S2).

### Enriched KEGG Pathways and GO Categories

Of the 13,075 genes for which expression data is given in GSE2361 that were analyzed in this work, only 3,729 (28.5%) had pathway information in KEGG and only 4,190 (32.0%) had tissue specificity data in PIR. For those genes with available pathway or tissue specificity data, those annotated with different KEGG pathways and PIR tissue specificity categories that were enriched (p<0.05) in the HK (TS) genes grouped according to *HKera* scores (see Methods) are presented in Table 3. Consistent with the notion that HK genes are expressed in a wide range of tissues, while TS genes are not, genes annotated with the PIR categories of “ubiquitous” and related terms were enriched only in the HK groups, while those annotated with tissue-specific expression (e.g. for liver and testis) were enriched only in the TS groups. In addition, apart from the seven pathways of molecular biology’s central functions that we have previously shown to be enriched in the HK_388_ set [28], several others indispensable to cells, such as DNA repair, energy production (oxidative phosphorylation), RNA degradation, and cell waste management (lysosome), were also enriched in the HK groups. Many disease- and infection-involved pathways were enriched in the HK groups, suggesting that many of the predicted HK genes are important for cell viability and that defects in these genes often lead to disease. In contrast, pathways involving biosynthesis, metabolism of sex and reproduction hormones, and metabolism of retinol and drugs (a reaction that takes place in liver [43]), were enriched in the TS groups.

**Table 3:** Number and percentage of genes annotated with the indicated KEGG pathway or PIR tissue specificity term enriched in different HK and TS gene sets.

Gene set^a^	enriched/annotatedgenes in this set	KEGG pathway or PIRtissue specificity term		Number of KEGG(PIR) genes	% of KEGG (PIR)genes in this set	p value^b^
HK_388_	KEGG: 270/276	hsa03010 Ribosome		84	94.1	2.0E-91
		hsa03050 Proteasome		42	92.9	1.8E-39
		hsa03040 Spliceosome		113	46.9	1.1E-32
		hsa03022 Basal transcription factors		33	69.7	1.7E-18
		hsa04120 Ubiquitin-mediated proteolysis		117	34.2	2.2E-17
		hsa00970 Aminoacyl-tRNA biosynthesis		30	63.3	2.2E-11
		hsa03020 RNA polymerase		11	100.0	3.7E-05
		hsa03420 Nucleotide excision repair		44	22.7	1.7E-02
	PIR: 26/61	(PIR) Ubiquitous		247	4.5	1.3E-04
		(PIR) Expressed ubiquitously		13	23.1	7.0E-03
		(PIR) Widely expressed		165	3.6	2.3E-02
		(PIR) Ubiquitously expressed		118	4.2	2.9E-02
HK_I_ (1∼2,000)	KEGG: 202/686	hsa00190 Oxidative phosphorylation		130	56.9	3.3E-29
		hsa05012 Parkinson's disease		128	57.0	7.6E-29
		hsa05010 Alzheimer's disease		163	46.0	6.2E-22
		hsa05016 Huntington's disease		180	43.9	1.3E-21
		hsa04722 Neurotrophin signaling pathway		124	33.1	7.5E-06
		hsa05110 Vibrio cholerae infection		56	42.9	4.9E-05
		hsa04142 Lysosome		117	29.9	1.1E-03
		hsa05120 Epithelial cell signaling inHelicobacter pylori infection		68	33.8	8.4E-03
		hsa05220 Chronic myeloid leukemia		75	32.0	1.5E-02
	PIR: 134/621	(PIR) Ubiquitous		247	28.3	9.7E-16
		(PIR) Ubiquitously expressed		118	23.7	4.1E-05
		(PIR) Widely expressed		165	21.2	4.5E-05
HK_II_ (2,001∼4,000)	KEGG: 51/595		hsa03018 RNA degradation	57	36.8	5.6E-04
			hsa04142 Lysosome	117	24.8	2.4E-02
	PIR: 49/685		(PIR) Ubiquitous	247	19.8	9.2E-05
			(PIR) Widely expressed	165	20.0	1.4E-03
			(PIR) Ubiquitously expressed	118	21.2	2.8E-03
HK_III_ (4,001∼6,000)	KEGG: 0/571		–	–	–	–
	PIR: 40/685		(PIR) Ubiquitous	247	16.2	1.8E-02
HK_IV_ (6,001∼7,761)	KEGG: 51/511		hsa04060 Cytokine-cytokine receptorinteraction interaction	262	19.5	6.5E-04
	PIR: 0/578		–	–	–	–
TS_I_ (7,762∼9,953)	KEGG: 110/638		hsa04080 Neuroactive ligand-receptorinteraction	256	21.1	9.7E-04
			hsa04060 Cytokine-cytokine receptorinteraction	262	20.6	3.5E-02
	PIR: 0/761		–	–	–	–
TS_II_ (9,954∼11,953)	KEGG: 77/728		hsa04610 Complement and coagulationcascades	69	49.3	3.9E-08
			hsa00830 Retinol metabolism	54	38.9	1.6E-02
			hsa00982 Drug metabolism	62	35.5	4.6E-02
			hsa00140 Steroid hormone biosynthesis	46	43.5	4.1E-03
			hsa00591 Linoleic acid metabolism	28	46.4	4.0E-02
	PIR_TS: 13/860		(PIR) Expressed by the liver and secretedinto the plasma	23	56.5	1.8E-05
TS_734_	KEGG: 12/222		hsa00140 Steroid hormone biosynthesis	46	26.1	4.4E-04
			hsa00150 Androgen and estrogenmetabolism	37	24.3	2.2E-02
	PIR_TS: 14/357		(PIR) Testis-specific	39	35.9	1.6E-07

a *HKera* score-sorted MR genes were divided into 4 HK sets (HK_I_-HK_IV_) and 2 TS sets (TS_I_ and TS_II_), each containing ∼2,000 genes (see Methods).

b The p values for the KEGG pathway were estimated using the Boferroni correction method by controlling the family-wide false discovery rate (FDR) under 5%. An additional criterion, gene number >10, was used to screen for genes enriched in the gene set with PIR tissue specificity annotations [37].

**Figure 5:**
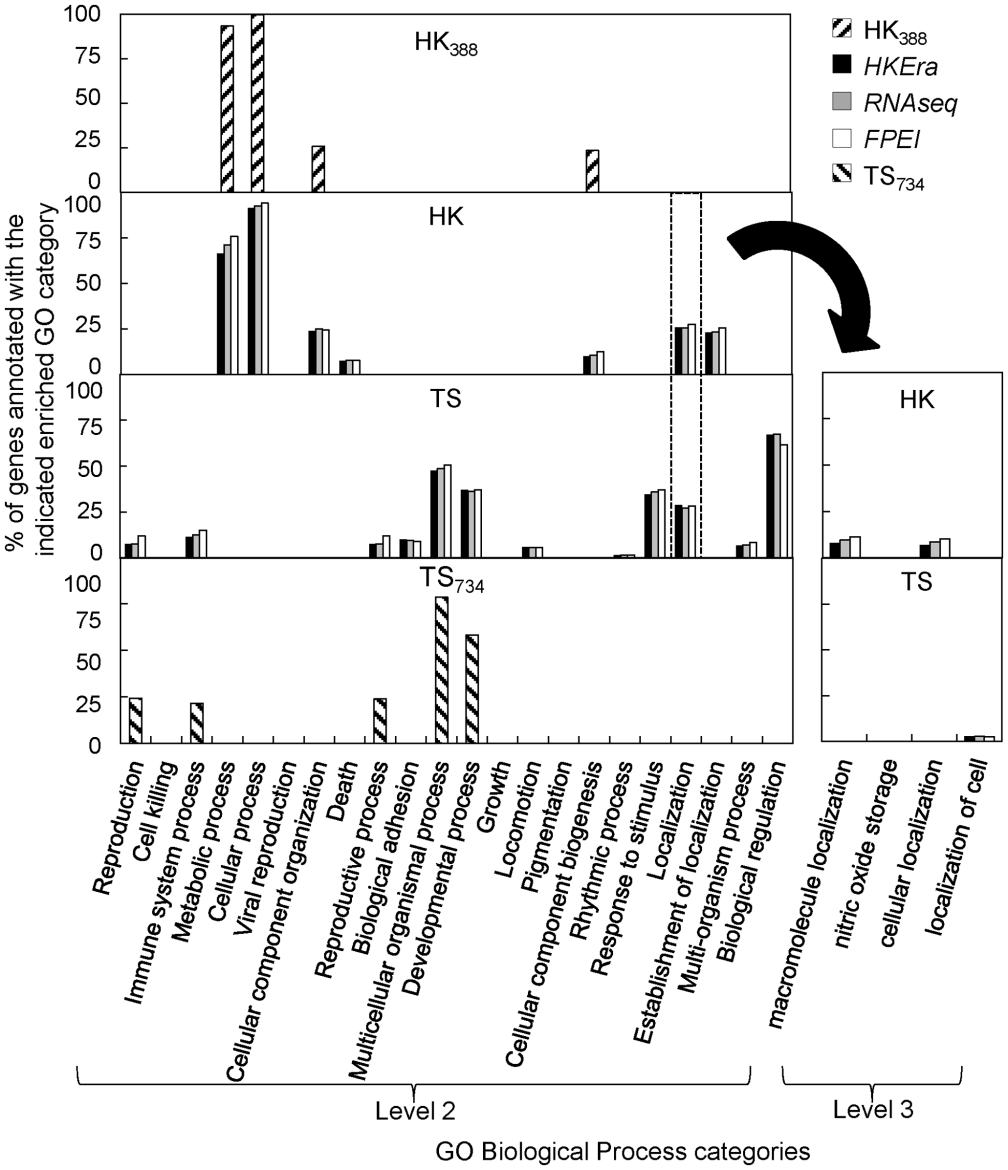
Percentage of genes annotated with the indicated GO term in different HK and TS sets. The figure shows the percentages of genes annotated with the indicated enriched GO category (determined using FDR-corrected p<0.05) in the indicated HK or TS gene set. Genes not associated with any enriched GO term were not used to compute the percentage. At GO’s level 2 (left graph), ‘Localization’ (GO: 0051179) was the only enriched BP category common to both the HK and TS sets; however, at the next level (level 3) of this category (right graph), the enriched GO terms for the two gene sets were different. Note that, “cellular localization (GO: 0051641)” and “macromolecule localization (GO: 0033036)” denote that a protein or macromolecule is transported to a specific location in a cell, while “localization of cell (GO: 0051674)” denotes that a cell is transported to a specific location [40]. Similar results were obtained for the analysis using GO Cellular Component (CC) (Figure S4 in File S1) and Molecular Function (MF) categories (Figure S5 in File S1).

Similarly, the GO terms showing up in the enrichment analysis were markedly different for HK and TS genes: this was especially evident for biological process (BP) (Figure 5) and cellular component (CC) (Figure S4 in File S1), but was also seen for molecular function (MF) (Figure S5 in File S1). Furthermore, with the exception of “binding” in MF (Figure S5 in File S1), those GO terms enriched in both HS and TS genes, i.e. “localization” in BP (Figure 5, left panel) and “structural molecule activity” in MF (Figure S5 in File S1), were separable at the next level of GO annotation (Figure 5, right panel and Figure S6 in File S1, respectively). This marked difference is generally in accordance with HK genes being involving in fundamental cellular processes and functional activities executed by various components of the cell and in different locations in the cell, and with TS genes being involving in regulation, immune, and other cellular responses, such as cell mobility.

## Discussion

**Figure 6:**
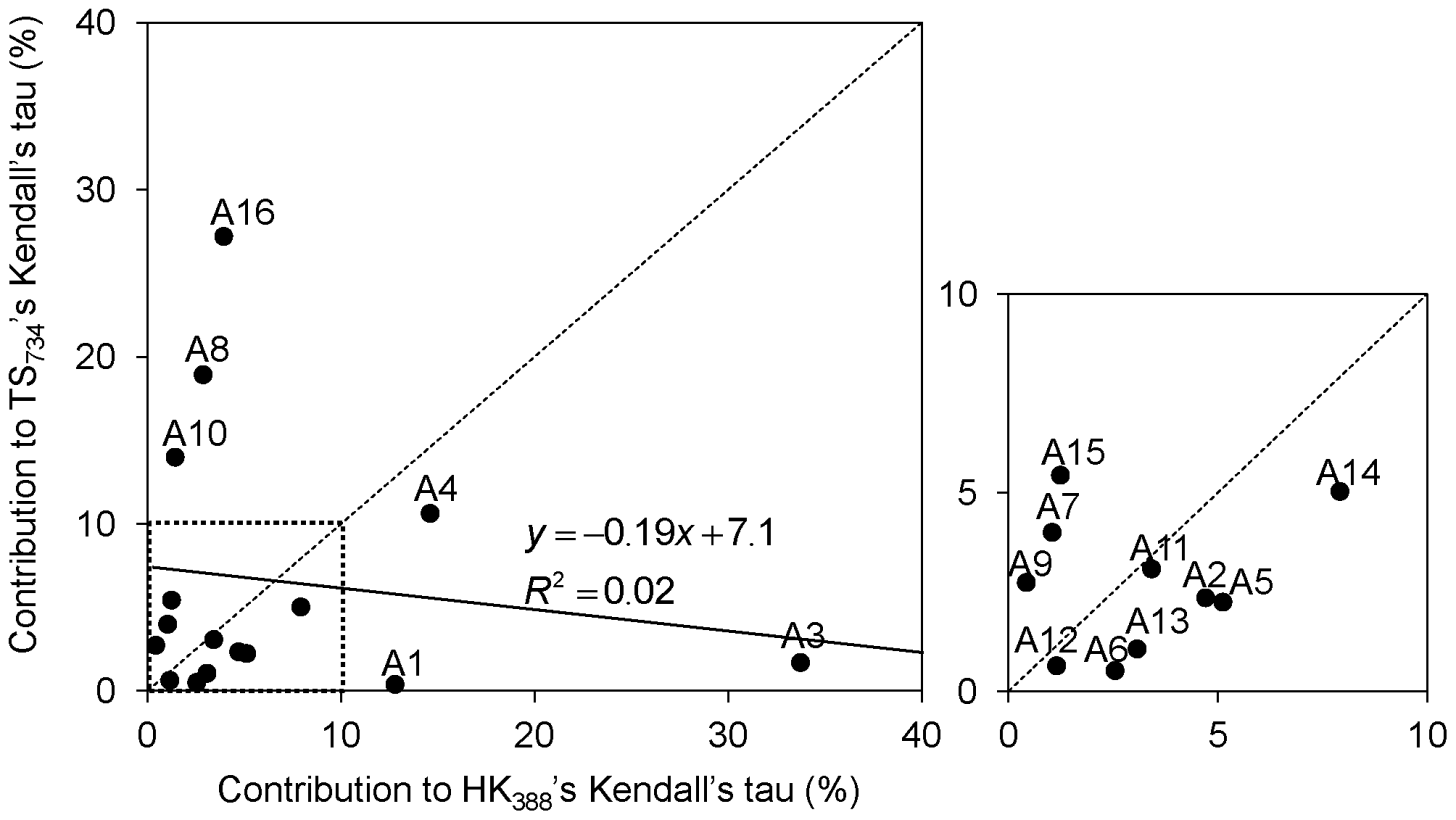
Contributions of the 16 tensor components to Kendall’s 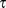 (TS_734_ vs. HK_388_). The equations for computing the 16 tensor components have been reported previously [28].

By definition, HK genes are expressed for functions that are common to all cells and TS genes are expressed for functions specific to certain types of cells. Consequently, the criterion of “ubiquitous expression” has commonly been employed to identify HK genes. Using microarray expression data, the number of HK genes identified has ranged from scores to a few hundreds [2], [10], [14], while, using *FPEI* predictions [15], it increases to ∼2,000. However, even this seemly large number of 2,000 is a gross underestimate compared to that of >6,000 obtained in experiments using *RNAseq* [6], so-called next generation sequencing capable of producing transcriptomes of a finer resolution than microarray technology [34]. In the present study, using the tensor structure of gene expression profiles, rather than expression levels or number of present calls, we showed that *HKera* was capable of identifying thousands of HK genes from microarray data with a good coverage of *RNAseq*-derived HK genes (Figure 4). Furthermore, compared to several other HK classifiers, *HKera* gave a significantly better performance against a number of benchmark HK sets derived from both microarray and RNAseq studies (Figure 2). It is noteworthy that the 16 ranking order-derived tensor components of gene expression profile were fairly orthogonal between the *TP* (HK_388_) and *TN* (TS_734_) data used to derive *HKera* (Figure 6), explaining its success. Indeed, *HKera* performed significantly better than SVM models trained on features used by the other HK classification methods compared, and including those features altogether achieved little, if any, improvement on *HKera*’s performance (Figure S7 in File S1). This is because the 16 attributes of *HKera* are much more significant features than those used by *Exp*, *TSI*, *FPEI*, *PCall*, and *Phy*, and had in fact captured almost all the information needed to classify the HK/TS genes in the benchmark training set, as indicated by the results of the information gain [44] analysis (Figure S8 in File S1). Leave-one(feature)-out analysis also showed that, of the 16 attributes (A1–A16), those with ranking presence (A1–A8) were slightly more important than those with ranking absence (A9–A16) in their impact on *HKera* performance, with A8 being the most significant feature (see Figures S1 and S2 in File S1 for explanations for the meaning of each of the 16 features). However, the differences were small, and leaving any feature out would all decrease, albeit not significantly, the accuracy of *HKera* predictions (Figure S9 in File S1). Since preservation of expression ranking order of HK genes has been previously observed using several different expression datasets and in data from different expression platforms [28], we can expect the *HKera* approach to be applicable to other large-scale gene expression data.

**Figure 7:**
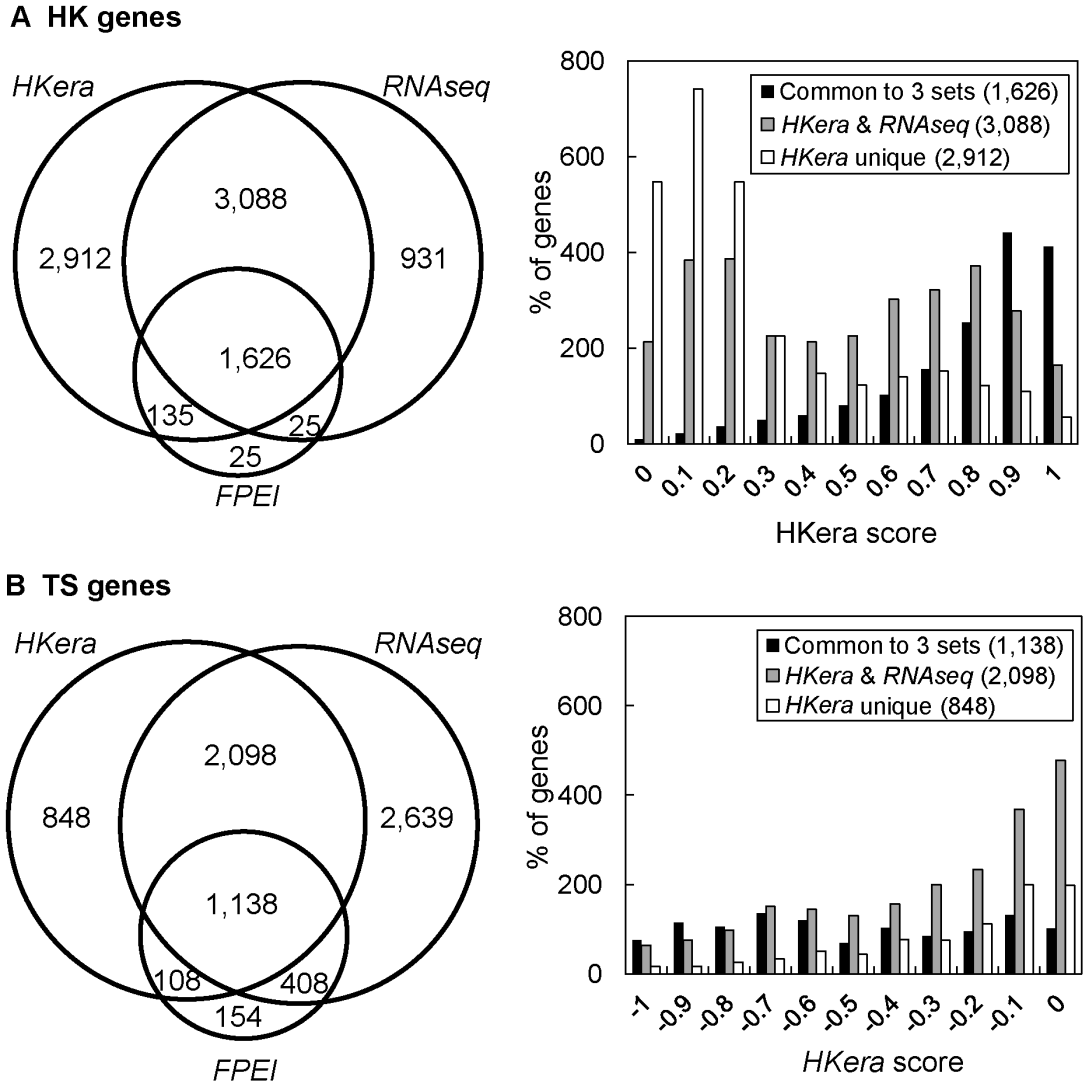
Common and unique HK/TS genes predicted by *HKera*, *RNAseq*, or *FPEI*, and their *HKera*-score distributions. Here, the canonical HK (HK_388_) and TS (TS_734_) genes were excluded, leaving only the MR genes for the analysis. Note that, whereas *FPEI* assigned a small subset of the transcriptome as HK or TS, *HKera* and *RNAseq* divided the transcriptome into two sets, i.e. those that were not predicted as HK genes were placed in the TS set.

It has been noted that the consensus between different HK gene sets identified by different methods, including those often used as benchmark, is not very good (10%–80%) [10], [15]. In comparison, the agreement between *HKera* and *RNAseq* was better: the percentage of genes designated as HK genes by *RNAseq* and predicted as such by *HKera* (using the threshold of a 0.0 *HKera* score) was 83.1%, while the converse coverage of *HKera*-predicted HK genes by *RNAseq* was 60.7% (Figure 7). The *HKera* scores (Figure 7, right panel) also showed that method-consensus genes (e.g. those common to the *HKera*, *RNAseq*, and *FPEI* sets or those only common to the *HKera* and *RNAseq* sets) generally had a better HK/TS-distinguishing *HKera* score than either *HKera*-unique or *RNAseq*-unique genes. Using a consensus from multiple prediction methods or a high *HKera* threshold would therefore be advisable practice for finding HK genes with high confidence. Nevertheless, many method-unique genes did have a good *HKera* score, some even as good as those of the benchmark genes (Figure 3), suggesting that different transcriptome-partitioning methods examine, to some extent, different features of the transcripome. These high *HKera*-score, method-unique genes are good candidates for novel HK genes.

Interestingly, although the sets of HK and TS genes classified by *HKera*, *RNAseq*, or *FPEI* were not highly concordant (Figure 7), they all contained essentially the same enriched GO terms, most of which would, in fact, have been captured by two much smaller gold-standard sets (HK_388_ and TS_734_, see Figure 5); moreover, the GO terms enriched in either HK or TS genes were highly complementary, such that, together, they covered most of the GO landscape (Figures 5, S4 (in File S1), and S5 (in File S1)). Perhaps the most telling observation for a distinct role of HK and TS genes is that, for BP, genes annotated as ‘cellular localization’ (“a localization process that takes place at the cellular level” [45]) were enriched in the HK genes, while those annotated as ‘localization of cell’ (“any process in which a cell is transported to, and/or maintained in, a specific location” [45]) were enriched in the TS genes (Figure 5, right panel). This was further demonstrated in a complete listing of HK- and TS-enriched CC terms (Table S3 and S4 in File S1, respectively), in which all GO levels were considered: namely, for example, genes annotated as ‘intracellular’ or ‘extracellular’ were enriched, respectively, in the HK genes or TS genes and, while genes annotated as ‘cytoplasm’ and ‘membrane’ were enriched in both the HK and TS genes, a more specific cell type (muscle for ‘cytoplasm’) or cell component (plasma membrane for ‘membrane’) was enriched in the TS genes.

In conclusion, we have developed a novel transcriptome partitioner and shown that it is superior to several other methods in reproducing ‘gold-standard’ HK gene sets. The large number (>7,000) of predicted HK genes is similar to that derived from RNAseq experiments and, as indicated by the enrichment analysis results, the human transcriptome can be partitioned into HK and TS gene sets that occupy distinct parts of the GO spectrum, reinforcing the notion that they have distinct cellular and functional roles.

## Supporting Information

File S1 Figure S1. Schematic illustrations of the 16 attributes used to derive *HKera*. (A) A schematic illustration of tissue-wide gene expression ranking (R) for a pair of genes and the three main contributing factors (Stableness (S), Co-expression (C) and Dispersion (D)) of its ranking order concordance and discordance [28]. The up and down arrows respectively point to increasing presence (+) and increasing absence (−) of the indicated variable. (B) Illustrations for each of the 16 attributes (A1–A16), which are products of a tensor operation on an equation that relates the expression ranking order (Kendall’s τ) and the three factors [28]. Figure S2. The distribution of attribute value for a gene pair and its tissue-wide expression and expression ranking profiles. (A) (Left) The fractional values for the presence (+) and absence (−) contribution of Stableness, Co-expression, Dispersion and Ranking as computed by tensor decomposition of gene expression ranking data [28], for a specific pair of genes (NCBI Entrez gene id 5143 and 55142). (Right) A wheel presentation of the composite values of the 16 attributes (A1–A16) used to derive *HKera*, showing that component A13 dominates for this gene pair. (B) Tissue-wide profile of gene expression levels (left) and rankings (right) for the two genes, showing that they have a high value on Stableness and Co-expression, but a low value on concordant rankings and Dispersion. N_I>0_ is the number of tissue pairs in which the rank of gene 5143 is higher than that of gene 55142, and N_I<0_ is the number of tissue pairs in which the rank of gene 5143 is lower than that of gene 55142. Note that there are a total 630 tissue pairs for 36 tissues, and ranking presence (R^+^) is low because N_I>0_ and N_I<0_ are about equal. Figure S3. Mean performance of *HKera* (SVM) and five other machine learning methods. Like *HKera*, models of the other machine learning methods were derived using the 16 tensor components of expression rankings as attributes. The performance was evaluated on the same training (A) and test (B) set data described in Methods. The performance represents the average of the results (for accuracy, recall, and precision; see Methods) obtained from five-fold cross validations. Error bars are standard deviations of the five-fold cross validation models. The six machine learning methods compared are: *HKera* (SVM), J48 (J48 decision tree, a logic-based algorithm), MLP (multilayer perception, a perceptron-based technique), OneR (one rule, a rule learning algorithm), NaïveBayes (a statistical learning algorithm), and KNN (k nearest neighbor, an instance-based learning algorithm). We used the Weka software (http://www.cs.waikato.ac.nz/ml/weka/) to derive these models. Figure S4. Percentage of genes annotated with the indicated CC term in different HK and TS sets. The figure shows the percentages of genes annotated with the indicated enriched GO category (determined using FDR-corrected p<0.05) in the indicated HK or TS gene set. Genes not associated with any enriched GO term were not used to compute the percentage. At GO’s level 2, the enriched GO terms for the two gene sets were different. Figure S5. Percentage of genes annotated with the indicated MF term in different HK and TS sets. The figure shows the percentages of genes annotated with the indicated enriched GO category (determined using FDR-corrected p<0.05) in the indicated HK or TS gene set. Genes not associated with any enriched GO term were not used to compute the percentage. At GO’s level 2, ‘Structural molecule activity’ (GO: 0005198) and ‘binding’ (GO: 0005488) were the two enriched MF categories common to both the HK and TS sets; however, at the next level (level 3) of these categories, the enriched GO terms for the two gene sets were mostly different (see Figure S6 in File S1 for the result of ‘Structural molecule activity’ at level 3; data not shown for ‘binding’). Figure S6. Percentage of genes annotated with ‘Structural molecule activity’ (GO:0005198) in different HK and TS sets. The figure shows the percentages of genes annotated with the indicated enriched GO category (determined using FDR-corrected p<0.05) in the indicated HK or TS gene set. Genes not associated with any enriched GO term were not used to compute the percentage. At GO’s level 3, the enriched GO terms of ‘structural molecule activity’ for the two gene sets were different. Figure S7. Mean performance of SVM*_HKera_*, SVM*_Conv_* and SVM*_All_*. The mean performance (average of accuracy, recall and precision rates from five-fold cross validation) of SVM models derived using different features: SVM*_HKera_* used *HKera* scores, SVM*_Conv_* used scores computed from the HK criteria (Table 1 in the main text) of the five conventional HK classification methods (*Exp*, *PCall*, *FPEI*, *TSI*, and *Phy*) compared in this study, and SVM*_All_* used all these scores. For *TSI* and *Phy*, the score was assigned to be the *TSI* index value and the *Phy* probability value, respectively; for *Exp*, *PCall* and *FPEI*, the score was the fraction of 36 tissues in which the gene in question was regarded as expressed by the method (e.g. expression intensity > = 200 for *Exp*, see Table 1). *RNAseq* was excluded because microarray gene expression data were used in this comparison. The performance was evaluated on the same training (A) and test (B) set data described in Methods. Error bars are standard deviations of the five-fold cross validation models. Figure S8. The information gain of the six HK classification features used to derive SVM*_All_*. The information gain, which ranges between 0 and 1 and can be computed based on theory of information entropy [44], is a measure of the capability of a feature to distinguish between HK class and TS class, based on the feature’s presence or absence in the HK_388_ and TS_734_ benchmark set (see Methods for the benchmark dataset and Figure S7 in File S1 for the derivation of SVM*_All_*). Error bars are standard deviations of the five-fold cross validation models. Figure S9. Leave-one(feature)-out accuracies of *HKera*. These accuracies were computed by leaving the indicated feature (one of the 16 tensor component attributes) out in *HKera* predictions of the training (A) and test (B) set data described in Methods. Arrow points to the accuracy of *HKera* using all of the 16 attributes (see Table 2). Error bars are standard deviations of the five-fold cross validation models. Table S1. Performance of *HKera* derived using different sets of reference genes. Table S3. Number of genes annotated with the indicated enriched cellular component GO terms in all levels in the HK genes predicted by *HKera.* Table S4. Number of genes annotated with the indicated enriched cellular component GO terms in all GO levels in the TS genes predicted by *HKera.*
(DOC)

File S2 Table S2. List of all 13,075 genes (in GSE2361) with their *HKera* score and GO, KEGG and PIR annotations. (This table is provided in a separate, Excel file).
(XLS)
