# Supplementary material for: Partitioning the Human Transcriptome Using HKera, a Novel Classifier of Housekeeping and Tissue-Specific Genes
Source: PLoS One. 2013 Dec 20;8(12):e83040. doi: 10.1371/journal.pone.0083040 (PMC3869736; doi:10.1371/journal.pone.0083040)
Supplement: File S1 — Figure S1. Schematic illustrations of the 16 attributes used to derive HKera. (A) A schematic illustration of tissue-wide gene expression ranking (R) for a pair of genes and the three main contributing factors (Stableness (S), Co-expression (C) and Dispersion (D)) of its ranking order concordance and discordance [28]. The up and down arrows respectively point to increasing presence (+) and increasing absence (−) of the indicated variable. (B) Illustrations for each of the 16 attributes (A1–A16), which are products of a tensor operation on an equation that relates the expression ranking order (Kendall’s τ) and the three factors [28]. Figure S2. The distribution of attribute value for a gene pair and its tissue-wide expression and expression ranking profiles. (A) (Left) The fractional values for the presence (+) and absence (−) contribution of Stableness, Co-expression, Dispersion and Ranking as computed by tensor decomposition of gene expression ranking data [28], for a specific pair of genes (NCBI Entrez gene id 5143 and 55142). (Right) A wheel presentation of the composite values of the 16 attributes (A1–A16) used to derive HKera, showing that component A13 dominates for this gene pair. (B) Tissue-wide profile of gene expression levels (left) and rankings (right) for the two genes, showing that they have a high value on Stableness and Co-expression, but a low value on concordant rankings and Dispersion. NI>0 is the number of tissue pairs in which the rank of gene 5143 is higher than that of gene 55142, and NI<0 is the number of tissue pairs in which the rank of gene 5143 is lower than that of gene 55142. Note that there are a total 630 tissue pairs for 36 tissues, and ranking presence (R+) is low because NI>0 and NI<0 are about equal. Figure S3. Mean performance of HKera (SVM) and five other machine learning methods. Like HKera, models of the other machine learning methods were derived using the 16 tensor components of expression rankings as attributes. The perform [file pone.0083040.s001.doc]

# Supporting information

## Figure S1. Schematic illustrations of the 16 attributes used to derive *HKera*. (A) A schematic illustration of tissue-wide gene expression ranking (R) for a pair of genes and the three main contributing factors (Stableness (S), Co-expression (C) and Dispersion (D)) of its ranking order concordance and discordance [28]. The up and down arrows respectively point to increasing presence (+) and increasing absence (-) of the indicated variable. (B) Illustrations for each of the 16 attributes (A1-A16), which are products of a tensor operation on an equation that relates the expression ranking order (Kendall’s τ) and the three factors [28].

## Figure S2. The distribution of attribute value for a gene pair and its tissue-wide expression and expression ranking profiles. (A) (Left) The fractional values for the presence (+) and absence (-) contribution of Stableness, Co-expression, Dispersion and Ranking as computed by tensor decomposition of gene expression ranking data [28], for a specific pair of genes (NCBI Entrez gene id 5143 and 55142). (Right) A wheel presentation of the composite values of the 16 attributes (A1-A16) used to derive *HKera*, showing that component A13 dominates for this gene pair. (B) Tissue-wide profile of gene expression levels (left) and rankings (right) for the two genes, showing that they have a high value on Stableness and Co-expression, but a low value on concordant rankings and Dispersion. N_I>0_ is the number of tissue pairs in which the rank of gene 5143 is higher than that of gene 55142, and N_I<0_ is the number of tissue pairs in which the rank of gene 5143 is lower than that of gene 55142. Note that there are a total 630 tissue pairs for 36 tissues, and ranking presence (R^+^) is low because N_I>0_ and N_I<0_ are about equal.

## Figure S3. Mean performance of *HKera* (SVM) and five other machine learning methods. Like *HKera*, models of the other machine learning methods were derived using the 16 tensor components of expression rankings as attributes. The performance was evaluated on the same training (A) and test (B) set data described in Methods. The performance represents the average of the results (for accuracy, recall, and precision; see Methods) obtained from five-fold cross validations. Error bars are standard deviations of the five-fold cross validation models. The compared six machine learning methods are: *HKera* (SVM), J48 (J48 decision tree, a logic-based algorithm), MLP (multilayer perception, a perceptron-based technique), OneR (one rule, a rule learning algorithm), NaïveBayes (a statistical learning algorithm), and KNN (k nearest neighbor, an instance-based learning algorithm). We used the Weka software ([**http://www.cs.waikato.ac.nz/ml/weka/**](http://www.cs.waikato.ac.nz/ml/weka/)) to derive these models.

## Figure S4. Percentage of genes annotated with the indicated CC term in different HK and TS sets. The figure shows the percentages of genes annotated with the indicated enriched GO category (determined using FDR-corrected p< 0.05) in the indicated HK or TS gene set. Genes not associated with any enriched GO term were not used to compute the percentage. At GO’s level 2, the enriched GO terms for the two gene sets were different.

## Figure S5. Percentage of genes annotated with the indicated MF term in different HK and TS sets. The figure shows the percentages of genes annotated with the indicated enriched GO category (determined using FDR-corrected p< 0.05) in the indicated HK or TS gene set. Genes not associated with any enriched GO term were not used to compute the percentage. At GO’s level 2, ‘Structural molecule activity’ (GO: 0005198) and ‘binding’ (GO: 0005488) were the two enriched MF categories common to both the HK and TS sets; however, at the next level (level 3) of these categories, the enriched GO terms for the two gene sets were mostly different (see Figure S6 in File S1 for the result of ‘Structural molecule activity’ at level 3; data not shown for ‘binding’).

## Figure S6. Percentage of genes annotated with ‘Structural molecule activity’ (GO:0005198) in different HK and TS sets. The figure shows the percentages of genes annotated with the indicated enriched GO category (determined using FDR-corrected p< 0.05) of in the indicated HK or TS gene set. Genes not associated with any enriched GO term were not used to compute the percentage. At GO’s level 3, the enriched GO terms of ‘structural molecule activity’ for the two gene sets were different.

## Figure S7. Mean performance of SVM*_HKera_*, SVM*_Conv_* and SVM*_All_*. The mean performances (average of accuracy, recall and precision rates from five-fold cross validation) of SVM models derived using different features: SVM*_HKera_* used *HKera* scores, SVM*_Conv_* used scores computed from the HK criteria (Table 1 in the main text) of the five conventional HK classification methods (*Exp*, *PCall*, *FPEI*, *TSI*, and *Phy*) compared in this study, and SVM*_All_* used all these scores. For *TSI* and *Phy*, the score was assigned to be the *TSI* index value and the *Phy* probability value, respectively; for *Exp*, *PCall* and *FPEI*, the score was the fraction of 36 tissues in which the gene in question was regarded as expressed by the method (e.g. expression intensity >=200 for *Exp*, see Table 1). *RNAseq* was excluded because microarray gene expression data were used in this comparison. The performance was evaluated on the same training (A) and test (B) set data described in Methods. Error bars are standard deviations of the five-fold cross validation models.

## Figure S8. The information gain of the six HK classification features used to derive SVM*_All_*. The information gain, which ranges between 0 and 1 and can be computed based on theory of information entropy [44], is a measure of the capability of a feature to distinguish between HK class and TS class, based on the feature’s presence or absence in the HK_388_ and TS_734_ benchmark set (see Methods for the benchmark dataset and Figure S7 in File S1 for the derivation of SVM*_All_*). Error bars are standard deviations of the five-fold cross validation models.

## Figure S9. Leave-one(feature)-out accuracies of *HKera*. These accuracies were computed by leaving the indicated feature (one of the 16 tensor component attributes) out in *HKera* predictions of the training (A) and test (B) set data described in Methods. The blue dashed horizontal lines were the accuracy rate of *HKera* with all of the 16 tensor component attributes (see Table 2)*.* Error bars are standard deviations of the five-fold cross validation models.

## Table S1. Performance of *HKera* derived using different sets of reference genes*.*

## Table S2 (in a separate, Excel file). List of all 13,075 genes (in GSE2361) with their *HKera* score and GO, KEGG and PIR annotations.

## Table S3. Number of genes annotated with the indicated enriched cellular component GO terms in all levels in the HK genes predicted by *HKera.*

## Table S4. Number of genes annotated with the indicated enriched cellular component GO terms in all GO levels in the TS genes predicted by *HKera.*

## Figure S1. Schematic illustrations of the 16 attributes used to derive *HKera*.

##
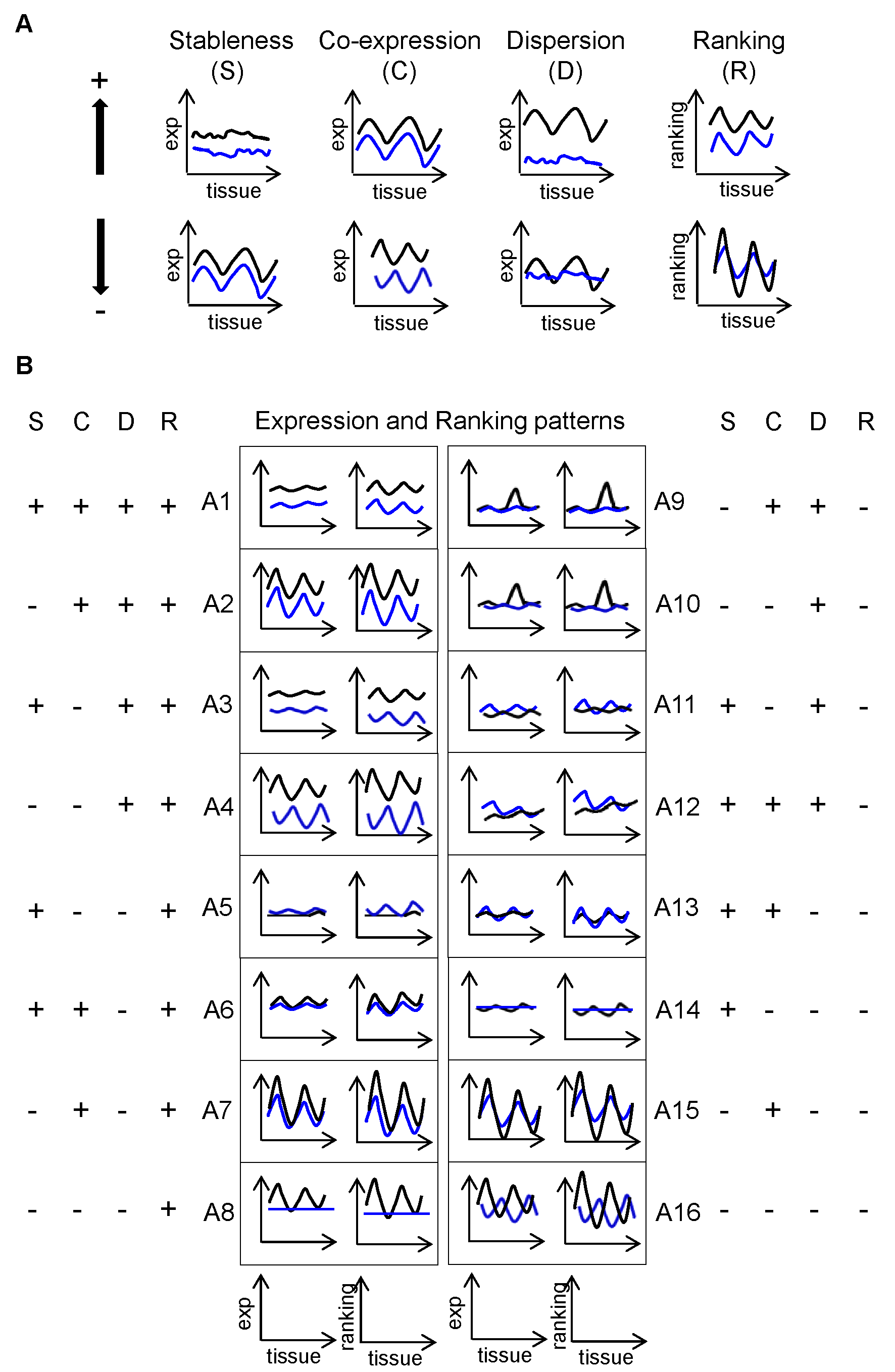


## Figure S2. The distribution of attribute value for a gene pair and its tissue-wide expression and expression ranking profiles.


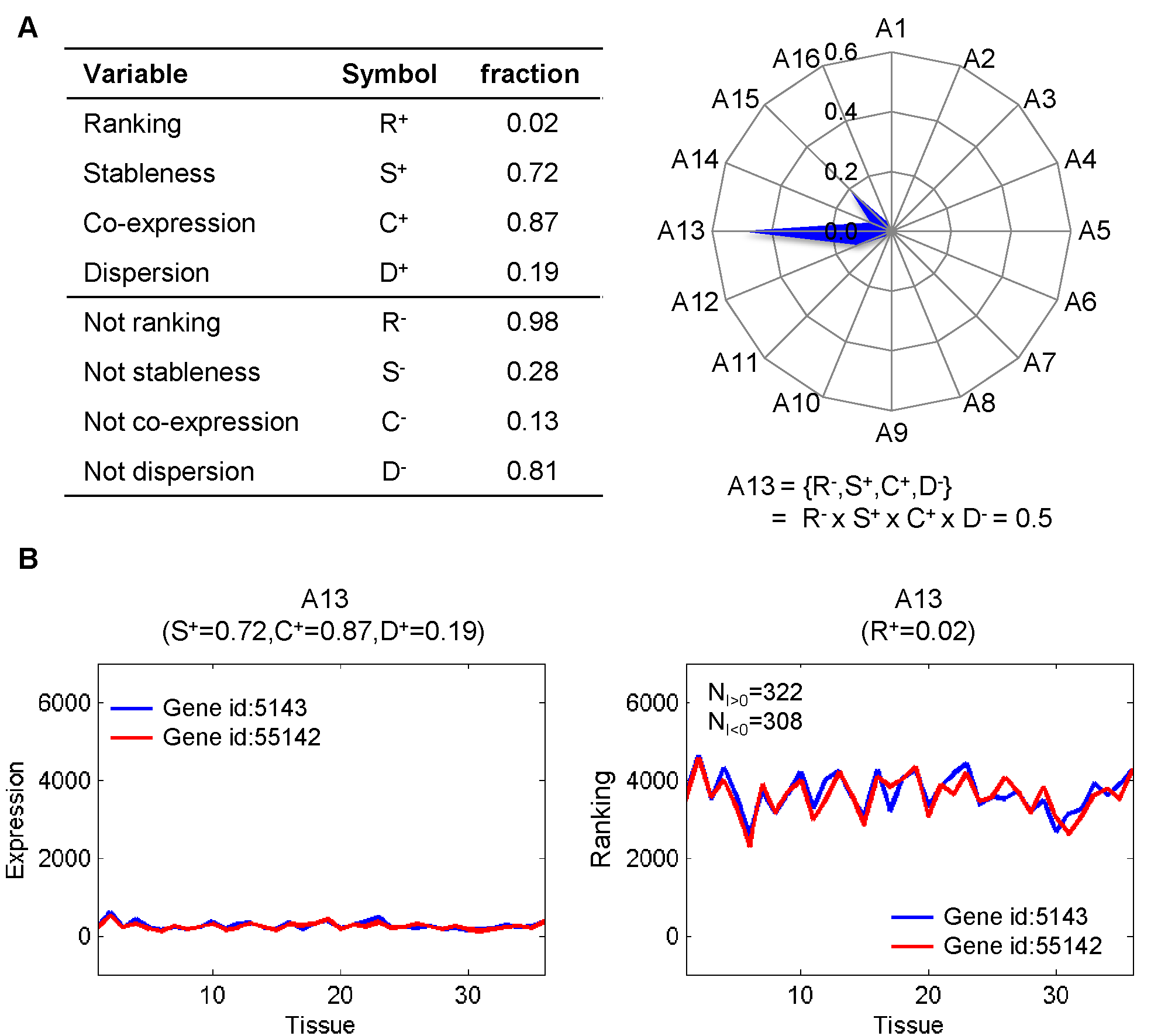


Figure S3. Mean performance of *HKera* (SVM) and five other machine learning methods.


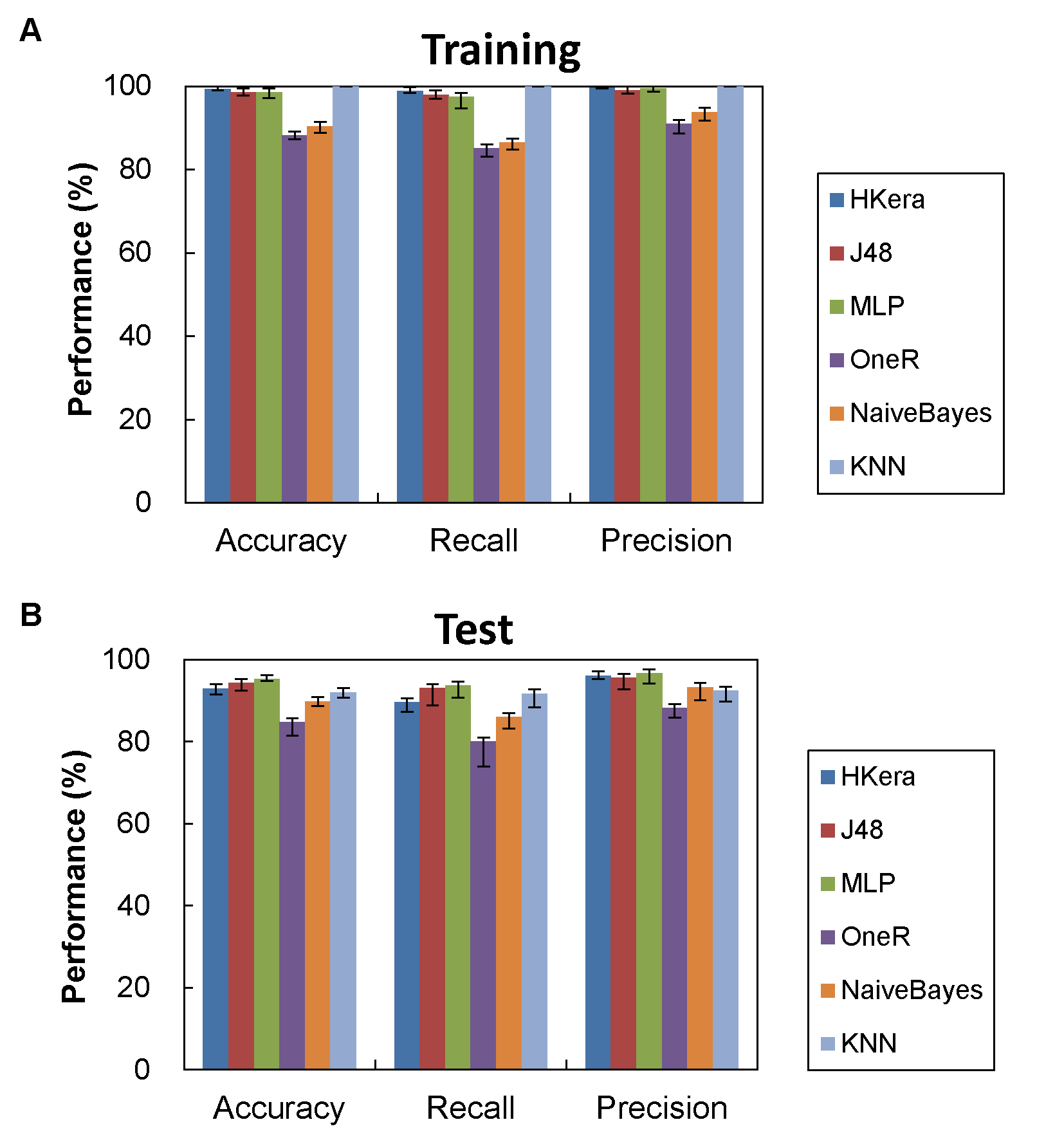


## Figure S4. Percentage of genes annotated with the indicated CC term in different HK and TS sets.


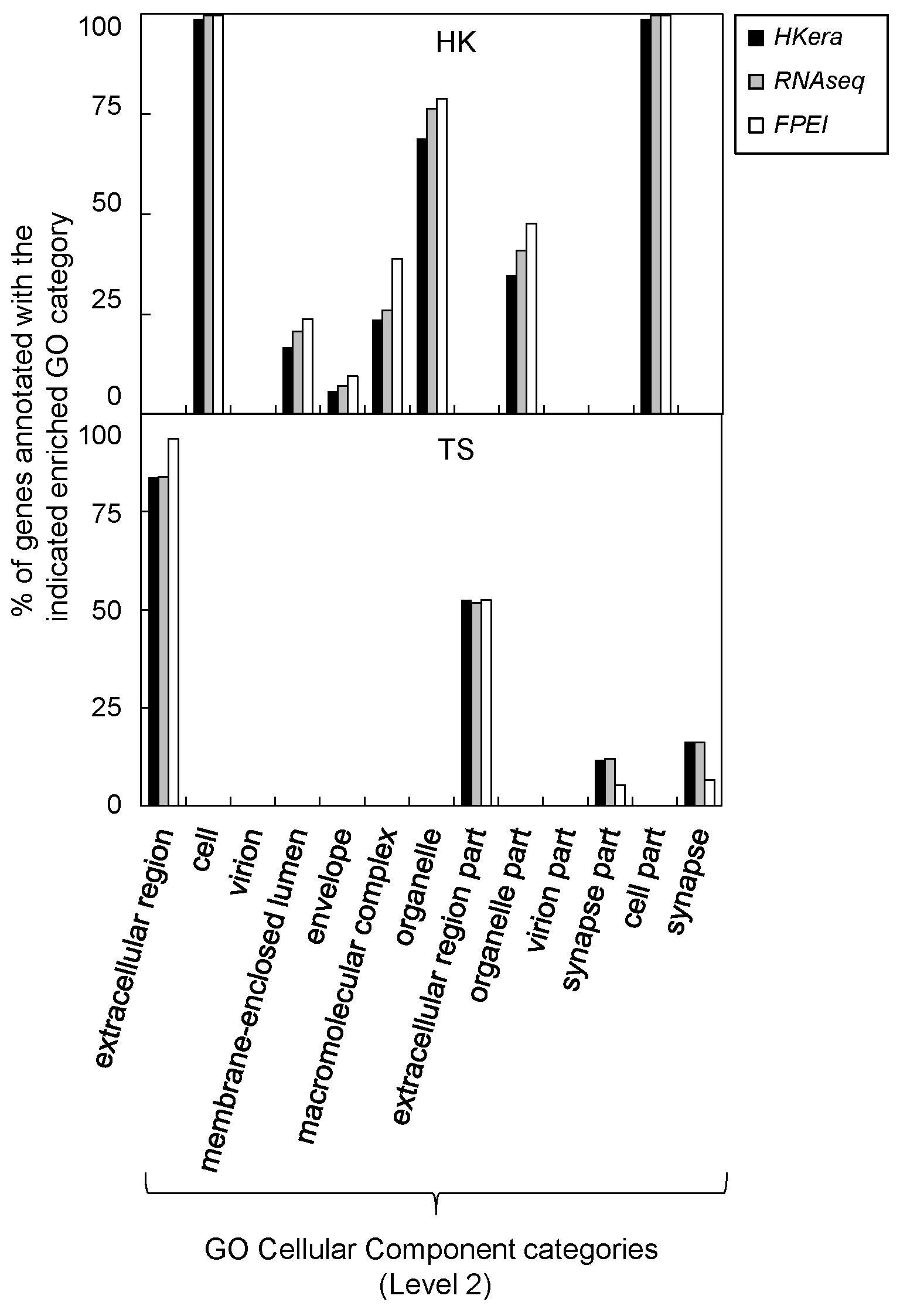


## Figure S5. Percentage of genes annotated with the indicated MF term in different HK and TS sets.

**
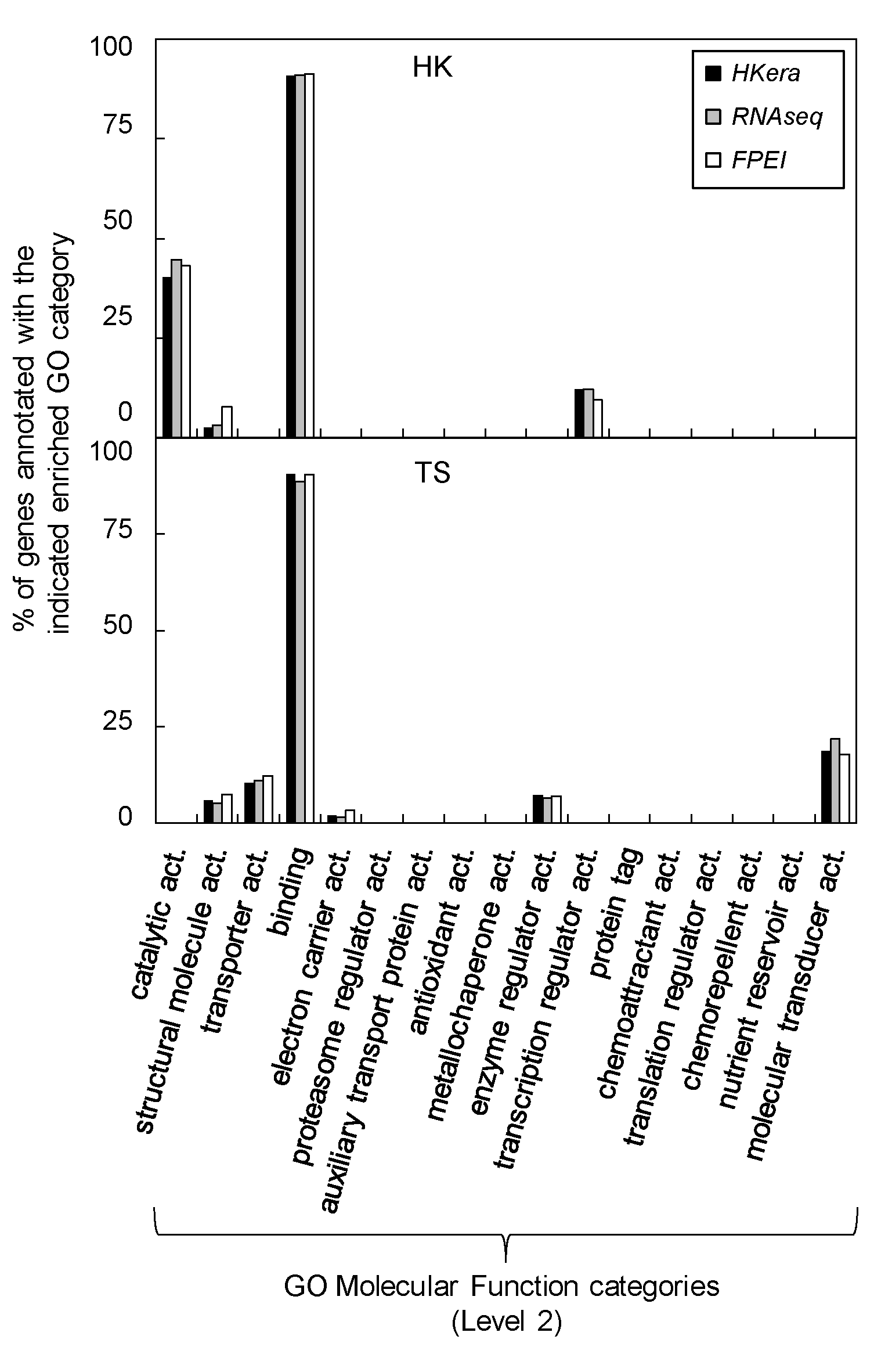
**

## Figure S6. Percentage of genes annotated with ‘Structural molecule activity’ (GO:0005198) in different HK and TS sets.

**
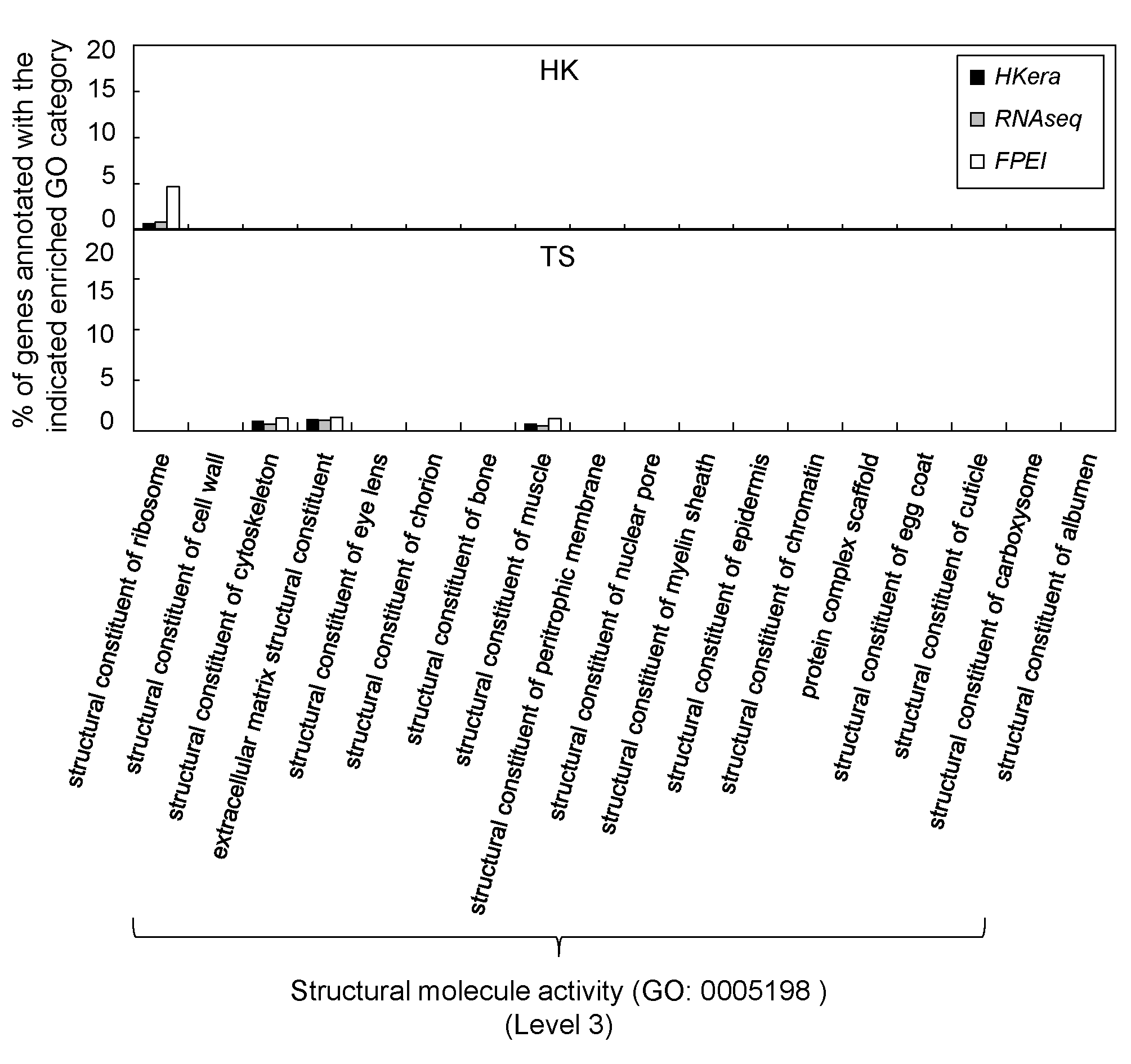
**

## Figure S7. Mean performance of SVM*_HKera_*, SVM*_Conv_* and SVM*_All_*.


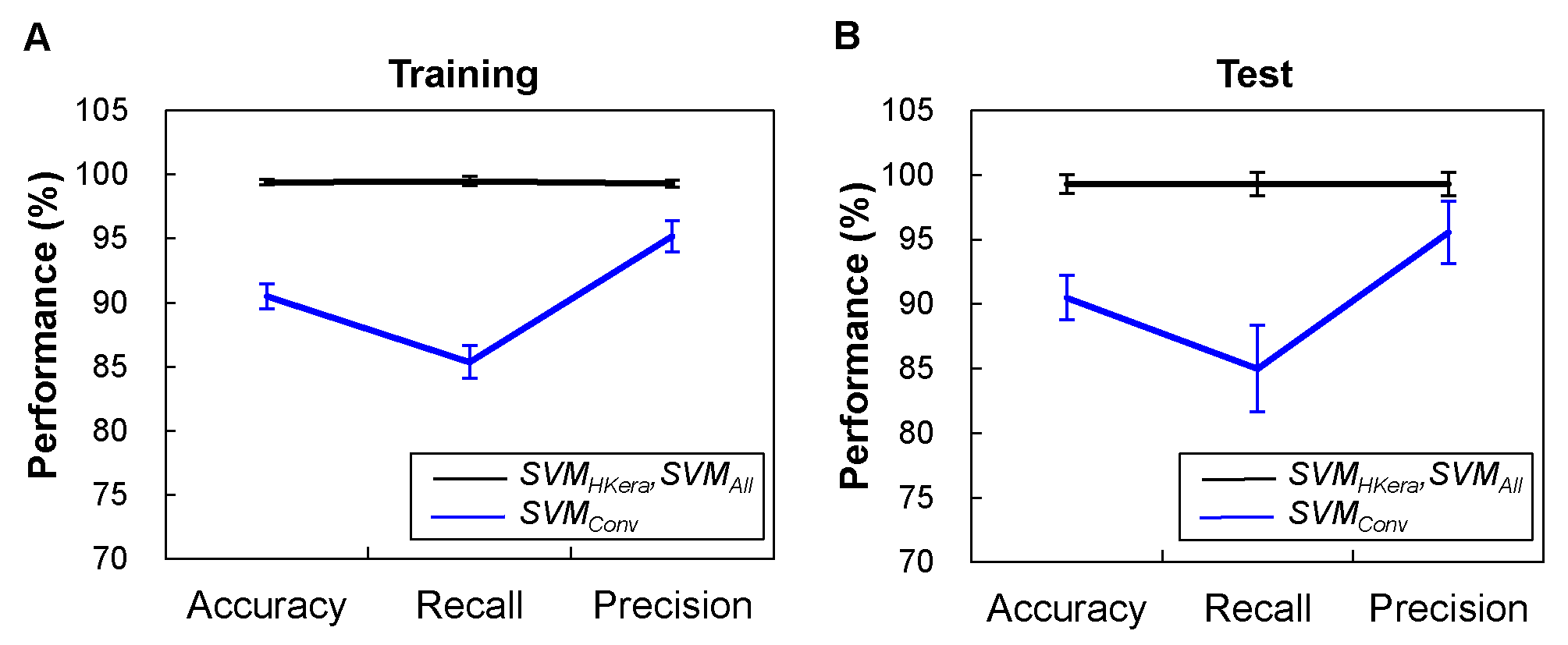


## Figure S8. The information gain of the six HK classification features used to derive SVM*_All_*.


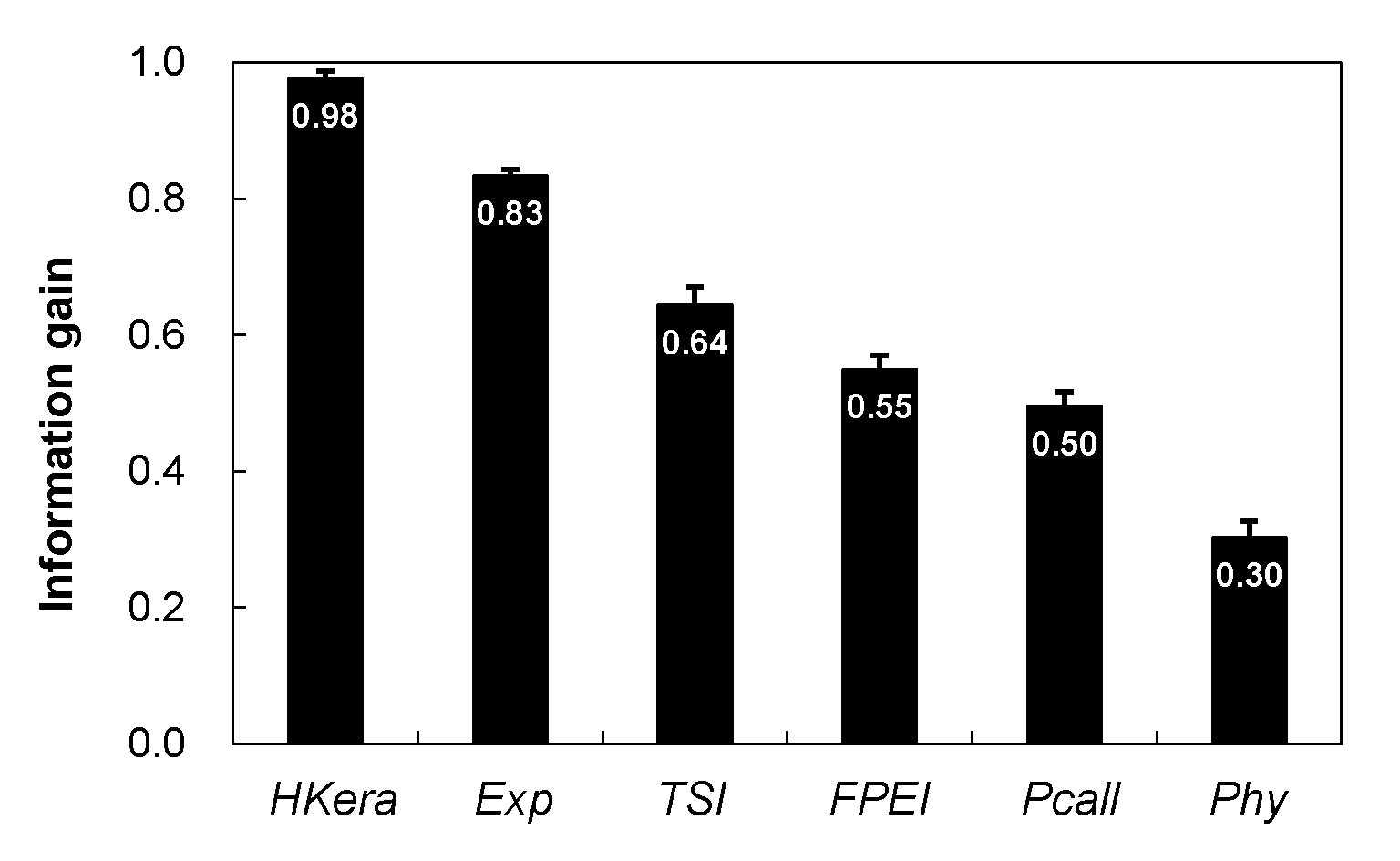


## Figure S9. Leave-one(feature)-out accuracies of *HKera*.

##
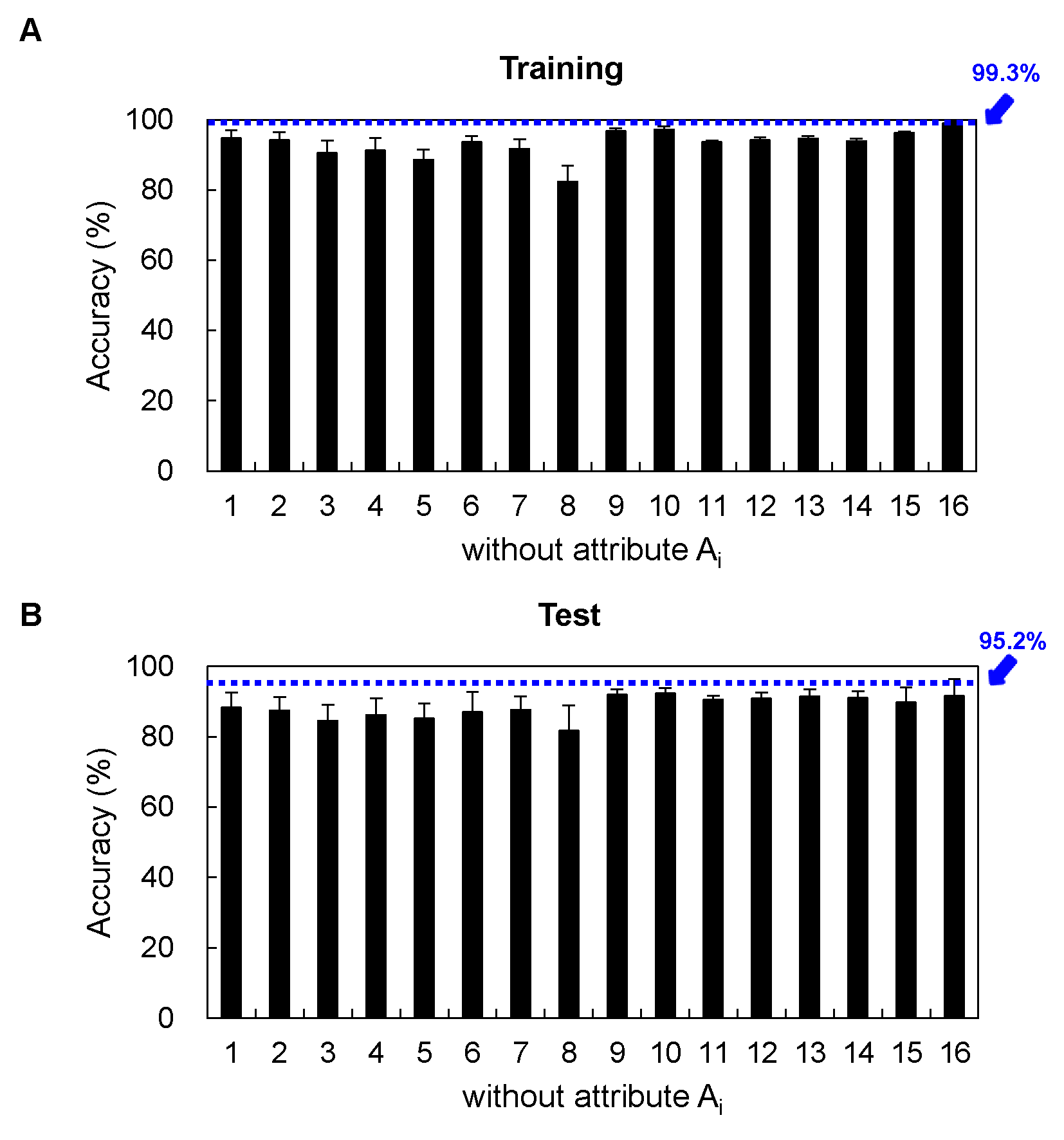


## Table S1. Performance of *HKera* derived using different sets of reference genes.

|  | **Accuracy ^a^** | |
| --- | --- | --- |
| **Reference gene set** | **Training** | **Test** |
| 50 HK genes | 99.8% | 96.5% |
| 25 HK + 25 TS | 99.6% | 95.2% |
| 50 TS genes | 99.2% | 92.9% |

^a^ Equation (4) in the main text.

**Table S3. Number of genes annotated with the indicated enriched cellular component GO terms in all levels in the HK genes predicted by *HKera.***

| **Class ^a^** | **GO ID** | **GO term** | **Number of GO genes** | **% of GO genes being HK genes** | **p value^b^** |
| --- | --- | --- | --- | --- | --- |
| Cytoplasm | GO:0000323 | lytic vacuole | 211 | 58.8 | 7.1E-05 |
| (20, 25%) | GO:0005737 | cytoplasm | 7,319 | 49.0 | 2.7E-93 |
|  | GO:0005739 | mitochondrion | 1,087 | 58.0 | 2.9E-31 |
|  | GO:0005740 | mitochondrial envelope | 419 | 59.9 | 4.0E-13 |
|  | GO:0005759 | mitochondrial matrix | 227 | 64.8 | 1.4E-10 |
|  | GO:0005764 | lysosome | 211 | 58.8 | 7.1E-05 |
|  | GO:0005768 | endosome | 315 | 54.0 | 7.7E-04 |
|  | GO:0005773 | vacuole | 252 | 59.1 | 1.7E-06 |
|  | GO:0005783 | endoplasmic reticulum | 960 | 49.1 | 1.0E-05 |
|  | GO:0005794 | Golgi apparatus | 872 | 56.7 | 7.0E-21 |
|  | GO:0005829 | cytosol | 1,330 | 51.3 | 8.1E-15 |
|  | GO:0016023 | cytoplasmic membrane-bounded vesicle | 550 | 52.0 | 1.5E-05 |
|  | **GO:0031410** ^c^ | **cytoplasmic vesicle** | 642 | 51.4 | **5.4E-06** |
|  | GO:0042470 | melanosome | 89 | 78.7 | 7.0E-10 |
|  | GO:0042645 | mitochondrial nucleoid | 31 | 87.1 | 6.9E-04 |
|  | GO:0044429 | mitochondrial part | 595 | 60.8 | 6.3E-22 |
|  | GO:0044431 | Golgi apparatus part | 294 | 60.2 | 5.6E-09 |
|  | GO:0044444 | cytoplasmic part | 4,895 | 50.7 | 4.1E-68 |
|  | GO:0048471 | perinuclear region of cytoplasm | 288 | 55.2 | 2.8E-04 |
|  | GO:0048770 | pigment granule | 89 | 78.7 | 7.0E-10 |
| Membrane | GO:0000139 | Golgi membrane | 186 | 59.7 | 1.2E-04 |
| (18, 22%) | GO:0005743 | mitochondrial inner membrane | 306 | 58.5 | 1.2E-07 |
|  | GO:0005746 | mitochondrial respiratory chain | 64 | 70.3 | 3.3E-03 |
|  | GO:0005747 | mitochondrial respiratory chain complex I | 42 | 73.8 | 3.7E-02 |
|  | GO:0005778 | peroxisomal membrane | 31 | 80.6 | 2.5E-02 |
|  | GO:0009898 | internal side of plasma membrane | 316 | 52.2 | 1.4E-02 |
|  | GO:0016469 | proton-transporting two-sector ATPase complex | 45 | 73.3 | 2.4E-02 |
|  | GO:0019866 | organelle inner membrane | 329 | 59.0 | 7.2E-09 |
|  | GO:0030964 | NADH dehydrogenase complex | 42 | 73.8 | 3.7E-02 |
|  | GO:0031090 | organelle membrane | 1,096 | 56.1 | 3.5E-25 |
|  | GO:0031300 | intrinsic to organelle membrane | 146 | 62.3 | 1.2E-04 |
|  | GO:0031301 | integral to organelle membrane | 123 | 63.4 | 3.9E-04 |
|  | GO:0031903 | microbody membrane | 31 | 80.6 | 2.5E-02 |
|  | GO:0031966 | mitochondrial membrane | 394 | 59.6 | 6.3E-12 |
|  | GO:0044455 | mitochondrial membrane part | 125 | 70.4 | 2.4E-08 |
|  | GO:0045259 | proton-transporting ATP synthase complex | 21 | 90.5 | 2.2E-02 |
|  | GO:0045271 | respiratory chain complex I | 42 | 73.8 | 3.7E-02 |
|  | GO:0070469 | respiratory chain | 75 | 69.3 | 9.5E-04 |

**Table S3.** (cont’)

| **Class^a^** | **GO ID** | **GO term** | **Number of GO genes** | **% of GO genes being HK genes** | **p value^b^** |
| --- | --- | --- | --- | --- | --- |
| Nucleus | GO:0005634 | nucleus | 5,077 | 48.6 | 2.2E-45 |
| (11, 14%) | GO:0005654 | nucleoplasm | 882 | 55.8 | 6.8E-19 |
|  | GO:0005730 | nucleolus | 698 | 58.0 | 3.0E-19 |
|  | GO:0009295 | nucleoid | 31 | 87.1 | 6.9E-04 |
|  | GO:0016585 | chromatin remodeling complex | 71 | 67.6 | 7.8E-03 |
|  | GO:0016604 | nuclear body | 168 | 62.5 | 9.5E-06 |
|  | GO:0016607 | nuclear speck | 103 | 63.1 | 5.1E-03 |
|  | GO:0017053 | transcriptional repressor complex | 41 | 75.6 | 1.7E-02 |
|  | GO:0031981 | nuclear lumen | 1,450 | 55.8 | 3.9E-33 |
|  | GO:0044428 | nuclear part | 1,822 | 54.7 | 3.0E-37 |
|  | GO:0044451 | nucleoplasm part | 555 | 56.9 | 1.0E-12 |
| Intracellular | GO:0005622 | intracellular | 10,995 | 46.7 | 1.3E-135 |
| (10, 12%) | GO:0015630 | microtubule cytoskeleton | 549 | 49.5 | 6.2E-03 |
|  | GO:0031967 | organelle envelope | 620 | 57.1 | 4.6E-15 |
|  | GO:0031980 | mitochondrial lumen | 227 | 64.8 | 1.4E-10 |
|  | GO:0043229 | intracellular organelle | 8,977 | 47.9 | 4.0E-108 |
|  | GO:0043231 | intracellular membrane-bounded organelle | 7,982 | 49.6 | 8.5E-126 |
|  | GO:0043232 | intracellular non-membrane-bounded organelle | 2,596 | 47.7 | 1.2E-14 |
|  | GO:0044424 | intracellular part | 10,624 | 46.9 | 7.9E-132 |
|  | GO:0044446 | intracellular organelle part | 4,225 | 51.1 | 3.6E-59 |
|  | GO:0070013 | intracellular organelle lumen | 1,779 | 56.7 | 4.4E-47 |
| Chromosome | GO:0000228 | nuclear chromosome | 162 | 60.5 | 2.9E-04 |
| (6, 8%) | GO:0000785 | chromatin | 200 | 62.5 | 3.3E-07 |
|  | GO:0000792 | heterochromatin | 41 | 75.6 | 1.7E-02 |
|  | GO:0005694 | chromosome | 460 | 55.0 | 1.1E-07 |
|  | GO:0044427 | chromosomal part | 386 | 56.0 | 3.8E-07 |
|  | GO:0044454 | nuclear chromosome part | 122 | 64.8 | 9.1E-05 |

**Table S3.** (cont’)

| **Class^a^** | **GO ID** | **GO term** | **Number of GO genes** | **% of GO genes being HK genes** | **p value^b^** |
| --- | --- | --- | --- | --- | --- |
| Others | **GO:0000267^c^** | **cell fraction** | **1,083** | **46.9** | **3.0E-03** |
| (15, 19%) | GO:0005623 | cell | 14,827 | 41.6 | 4.9E-38 |
|  | GO:0012505 | endomembrane system | 782 | 52.6 | 1.1E-09 |
|  | GO:0031974 | membrane-enclosed lumen | 1,856 | 56.5 | 3.0E-48 |
|  | GO:0031975 | envelope | 622 | 57.1 | 4.4E-15 |
|  | **GO:0031982^c^** | **vesicle** | **670** | **52.1** | **2.6E-07** |
|  | GO:0031988 | membrane-bounded vesicle | 568 | 52.6 | 1.3E-06 |
|  | GO:0032991 | macromolecular complex | 3,155 | 46.7 | 2.0E-13 |
|  | GO:0043226 | organelle | 8,989 | 47.9 | 7.1E-109 |
|  | GO:0043227 | membrane-bounded organelle | 7,989 | 49.6 | 3.1E-127 |
|  | GO:0043228 | non-membrane-bounded organelle | 2,596 | 47.7 | 1.2E-14 |
|  | GO:0043233 | organelle lumen | 1,820 | 56.5 | 2.8E-47 |
|  | GO:0043234 | protein complex | 2,588 | 46.9 | 4.8E-11 |
|  | GO:0044422 | organelle part | 4,251 | 51.0 | 4.4E-59 |
|  | GO:0044464 | cell part | 14,826 | 41.6 | 3.7E-38 |

1. Classes were manually curated based on results using CateGOrizer (<http://www.animalgenome.org/tools/catego/>) and the following three steps: 1) for identical GO terms appearing in more than one level, only the highest level was retained; 2) when there were more than 5, related GO terms were grouped into a single class (e.g. ‘extracellular space’, ‘extracellular matrix’, and ‘extracellular region’ were grouped into the class of ‘extracellular’); 3) the rest of the enriched GO terms were put into the “Others” class. The values in parentheses are the number of enriched GO terms in the indicated class and this number expressed as a percentage of the total number of enriched GO terms.
2. A GO term was considered ‘enriched’ if the p value was < 0.05. In this table, only those satisfying this criterion were included.
3. Boldface indicates GO terms shared by both HK and TS genes (see Table S4 for results of TS genes).

## Table S4. Number of genes annotated with the indicated enriched cellular component GO terms in all GO levels in the TS genes predicted by *HKera.*

| **Class^a^** | **GO ID** | **GO term** | **Number of GO genes** | **% of GO genes being TS genes** | **p value^b^** |
| --- | --- | --- | --- | --- | --- |
| Cytoplasm | GO:0030016 | myofibril | 111 | 47.7 | 9.9E-06 |
| (Muscle) | GO:0030017 | sarcomere | 98 | 48.0 | 6.2E-05 |
| (8, 17%) | GO:0030141 | secretory granule | 180 | 36.7 | 1.7E-02 |
|  | **GO:0031410**^c^ | **cytoplasmic vesicle** | 642 | 29.6 | **1.5E-02** |
|  | GO:0031674 | I band | 55 | 50.9 | 1.0E-02 |
|  | GO:0043292 | contractile fiber | 121 | 47.9 | 1.6E-06 |
|  | GO:0044433 | cytoplasmic vesicle part | 187 | 38.0 | 2.0E-03 |
|  | GO:0044449 | contractile fiber part | 113 | 48.7 | 2.2E-06 |
| Membrane | GO:0005886 | plasma membrane | 3,777 | 30.3 | 8.7E-35 |
| (Plasma) | GO:0005887 | integral to plasma membrane | 1,188 | 42.1 | 2.9E-52 |
| (9, 20%) | GO:0009897 | external side of plasma membrane | 170 | 42.4 | 1.1E-05 |
|  | GO:0016020 | membrane | 7,266 | 24.3 | 3.8E-04 |
|  | GO:0016324 | apical plasma membrane | 133 | 42.9 | 2.6E-04 |
|  | GO:0031226 | intrinsic to plasma membrane | 1,215 | 42.1 | 7.1E-54 |
|  | GO:0031253 | cell projection membrane | 65 | 46.2 | 4.7E-02 |
|  | GO:0034702 | ion channel complex | 205 | 35.6 | 1.9E-02 |
|  | GO:0044459 | plasma membrane part | 2,203 | 36.9 | 5.2E-59 |
| Cell projection | GO:0014069 | postsynaptic density | 71 | 47.9 | 5.3E-03 |
| (Neuron) | GO:0030424 | axon | 159 | 42.1 | 4.8E-05 |
| (6, 20%) | GO:0030425 | dendrite | 163 | 38.7 | 4.0E-03 |
|  | GO:0042995 | cell projection | 697 | 35.3 | 4.9E-12 |
|  | GO:0043005 | neuron projection | 342 | 40.6 | 5.3E-11 |
|  | GO:0044463 | cell projection part | 234 | 35.5 | 5.9E-03 |
| Extracellular | GO:0005576 | extracellular region | 2,010 | 33.1 | 6.8E-29 |
| (9, 13%) | GO:0005578 | proteinaceous extracellular matrix | 320 | 39.7 | 4.9E-09 |
|  | GO:0005581 | collagen | 35 | 65.7 | 2.7E-04 |
|  | GO:0005615 | extracellular space | 685 | 46.9 | 1.6E-43 |
|  | GO:0031012 | extracellular matrix | 345 | 39.7 | 6.4E-10 |
|  | GO:0032994 | protein-lipid complex | 35 | 60.0 | 7.3E-03 |
|  | GO:0034358 | plasma lipoprotein particle | 35 | 60.0 | 7.3E-03 |
|  | GO:0044420 | extracellular matrix part | 117 | 47.9 | 3.3E-06 |
|  | GO:0044421 | extracellular region part | 960 | 43.5 | 2.1E-47 |

**Table S4.** (cont’)

| **Class^a^** | **GO ID** | **GO term** | **Number of GO genes** | **% of GO genes being TS genes** | **p value^b^** |
| --- | --- | --- | --- | --- | --- |
| Others | **GO:0000267**^c^ | **cell fraction** | 1,083 | 36.1 | **4.9E-23** |
| (14, 30%) | GO:0005624 | membrane fraction | 809 | 37.2 | 2.2E-19 |
|  | GO:0005625 | soluble fraction | 313 | 32.6 | 3.1E-02 |
|  | GO:0005626 | insoluble fraction | 839 | 37.1 | 8.2E-20 |
|  | GO:0005856 | cytoskeleton | 1,381 | 27.7 | 1.4E-03 |
|  | GO:0009986 | cell surface | 348 | 37.1 | 6.6E-07 |
|  | GO:0015629 | actin cytoskeleton | 269 | 37.9 | 1.2E-05 |
|  | GO:0030054 | cell junction | 518 | 34.9 | 6.4E-08 |
|  | **GO:0031982**^c^ | **vesicle** | 670 | 29.6 | **1.1E-02** |
|  | GO:0043025 | cell soma | 168 | 38.1 | 5.8E-03 |
|  | GO:0044430 | cytoskeletal part | 952 | 28.3 | 1.3E-02 |
|  | GO:0044456 | synapse part | 246 | 37.8 | 6.2E-05 |
|  | GO:0045177 | apical part of cell | 179 | 41.3 | 2.4E-05 |
|  | GO:0045202 | synapse | 355 | 36.6 | 1.4E-06 |

1. Classes were manually curated based on results using CateGOrizer (<http://www.animalgenome.org/tools/catego/>) and the following three steps: 1) for identical GO terms appearing in more than one level, only the highest level was retained; 2) when there were more than 5, related GO terms were grouped into a single class (e.g. ‘extracellular space’, ‘extracellular matrix’, and ‘extracellular region’ were grouped into the class of ‘extracellular’); 3) the rest of the enriched GO terms were put into the “Others” class. The values in parentheses are the number of enriched GO terms in the indicated class and this number expressed as a percentage of the total number of enriched GO terms.
2. A GO term was considered ‘enriched’ if the p value was < 0.05. In this table, only those satisfying this criterion were included.
3. Boldface indicates GO terms shared by both HK and TS genes (see Table S3 for results of HK genes).
